# Hepatocyte-derived FGL1 accelerates liver metastasis and tumor growth by inhibiting CD8^+^ T and NK cells

**DOI:** 10.1172/jci.insight.173215

**Published:** 2024-05-23

**Authors:** Fengjia Xi, Haoyu Sun, Hui Peng, Zhexiong Lian, Haiming Wei, Zhigang Tian, Rui Sun, Yongyan Chen

**Affiliations:** 1 Key Laboratory of Immune Response and Immunotherapy, the Institute of Immunology, Biomedical Sciences and Health Laboratory of Anhui Province, Center for Advanced Interdisciplinary Science and Biomedicine of IHM, School of Basic Medical Sciences, Division of Life Sciences and Medicine, University of Science and Technology of China, Hefei, China.; 2 Research Unit of NK Cell Study, Chinese Academy of Medical Sciences, Hefei, China.

**Keywords:** Hepatology, Immunology, Cancer immunotherapy, Liver cancer

## Abstract

Fibrinogen-like protein 1 (FGL1) contributes to the proliferation and metabolism of hepatocytes; however, as a major ligand of the immune checkpoint, its role in the liver regional immune microenvironment is poorly understood. Hepatocytes specifically and highly expressed FGL1 under normal physiological conditions. Increases in hepatic CD8^+^ T and NK cell numbers and functions were found in *Fgl1*-deficient (*Fgl1*^–/–^) mice, but not in the spleen or lymph node, similar to findings in anti-FGL1 mAb–treated wild-type mice. Furthermore, *Fgl1* deficiency or anti-FGL1 mAb blockade restrained liver metastasis and slowed the growth of orthotopic tumors, with significantly prolonged survival of tumor-bearing mice. Tumor-infiltrating hepatic CD8^+^ T and NK cells upregulated the expression of lymphocyte activation gene-3 (LAG-3) and exhibited stronger antitumor activities after anti-FGL1 treatment. The antitumor efficacy of FGL1 blockade depended on cytotoxic T lymphocytes and NK cells, demonstrated by using a cell-deficient mouse model and cell transfer in vivo. In vitro, FGL1 directly inhibited hepatic T and NK cells related to the receptor LAG-3. In conclusion, hepatocyte-derived FGL1 played critical immunoregulatory roles in the liver and contributed to liver metastasis and tumor growth by inhibiting CD8^+^ T and NK cell functions via the receptor LAG-3, providing a new strategy for liver cancer immunotherapy.

## Abstract 2

Hepatocyte-derived FGL1 plays critical immunoregulatory roles in the liver and contributes to liver metastasis and tumor growth.

## Graphical abstract

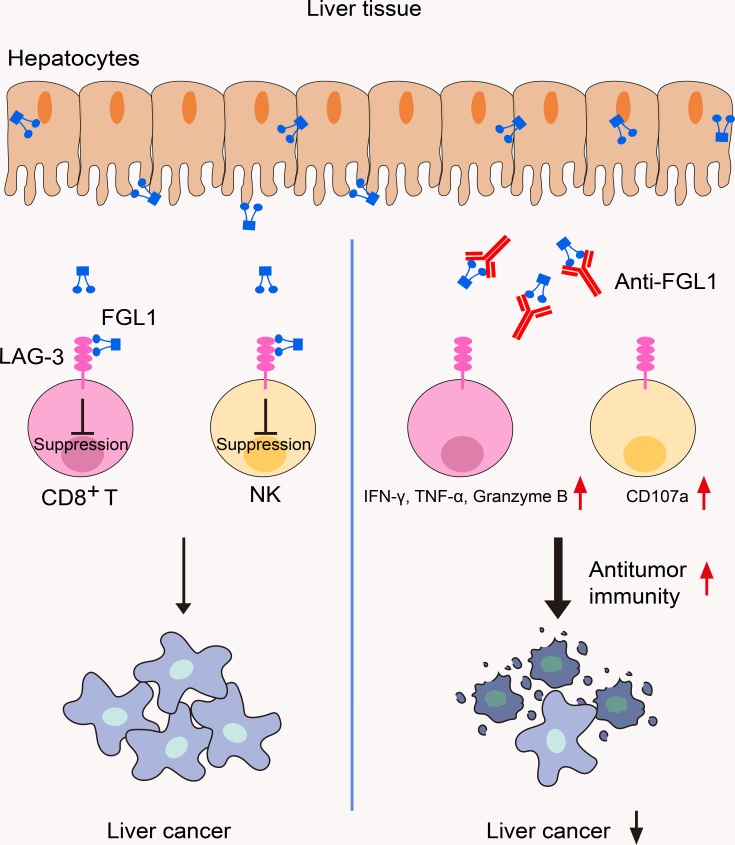

## Introduction

Primary liver cancer was the sixth most common cancer and the third leading cause of cancer death worldwide in 2020, with approximately 906,000 new cases and 830,000 deaths (1). In addition to being highly susceptible to primary tumors, the liver is also a common site of tumor metastasis, including a wide variety of malignancies of colorectal cancer (CRC), pancreatic cancer, melanoma, lung cancer, and breast cancer (2). In the last 5 years, immunotherapy with checkpoint blockade has changed the management of hepatocellular carcinoma (HCC). Clinical trials have shown that blocking cytotoxic T lymphocyte-associated antigen-4 (CTLA-4) or programmed cell death-1 (PD-1)/programmed death ligand 1 (PD-L1) is active, tolerable, and clinically beneficial against advanced HCC, but the highest response rate is approximately 20%. An increased response rate of approximately 30% is observed with the dual blockade of CTLA-4 and PD-1/PD-L1 (3). Liver tumor-infiltrating lymphocytes also express higher levels of lymphocyte activation gene-3 (LAG-3), T cell immunoglobulin-3 (TIM-3), and T cell immunoglobulin and ITIM domain (TIGIT), providing support for exploring the next generation of checkpoints in liver cancer immunotherapy (4, 5).

Fibrinogen-like protein 1 (FGL1), a ligand of the inhibitory receptor LAG-3, can induce LAG-3 clustering and has recently been considered a checkpoint for tumor immunotherapy (6–8). These findings attracted our attention to the roles of FGL1 in the liver, since FGL1 was initially termed a hepatokine and is specifically expressed in liver tissue under normal conditions and in HCC cell lines (9). FGL1 acts as a hepatic growth factor that can promote hepatocyte proliferation and prevent hepatocyte apoptosis and oxidative stress when the liver is injured (10, 11). Excessive FGL1 causes hepatic lipid accumulation, inflammation, and insulin resistance, leading to nonalcoholic fatty liver disease (NAFLD), obesity, and type 2 diabetes (12–14). Studies in the last decade have focused on the roles of FGL1 in the liver, an organ with amazing regenerative ability and critical metabolic function.

Noticeably, the liver is a unique immune-tolerant organ (15). When *Fgl1* was deficient, increased central memory-like CD8^+^ T cells and decreased B cells were observed in peripheral blood, and autoimmune disease developed in aged mice (6). Small extracellular vesicles expressing FGL1 and PD-L1 could reestablish immune tolerance and significantly alleviate immune rejection in a heart allograft model (16). Consistently, FGL1 recombinant protein administered intraperitoneally significantly attenuated T cell–mediated autoimmune disease arthritis (17). These findings suggest that liver-derived FGL1 is involved in host immune homeostasis as an important immunosuppressive molecule. However, the precise role and mechanism of FGL1 in the liver regional immune microenvironment remain unclear.

Increased FGL1 was detected in HCC, including hepatitis B virus–related (HBV-related) HCC, and was related to a poor prognosis (18–20). HCC patients with higher levels of FGL1 on circulating tumor cells showed a higher proportion of advanced tumor node and metastasis stage as well as distant metastases and worse postoperative survival (21). Using a mouse subcutaneous tumor model of hepatoma cells, Hepa1-6, it was found that *Fgl1* deficiency inhibited tumor growth (6). Reducing FGL1 levels by oxysophocarpine or aspirin treatment sensitized HCC cells to anti–LAG-3 or anti–PD-L1 immunotherapy in the subcutaneous tumor model (22, 23). Conversely, increased HCC tumor growth induced by diethylnitrosamine was observed in *Fgl1*-deficient mice compared with control wild-type mice (24). These findings indicated that the roles of FGL1 might be complex during liver tumor development and progression. Whether FGL1, a new ligand of LAG-3, has therapeutic potential for liver cancer still needs to be investigated.

In this study, we found that hepatocyte-derived FGL1 was especially associated with the liver regional immune microenvironment under normal physiological conditions. By using mouse models of liver metastasis and orthotopic liver cancer, it was demonstrated that *Fgl1* deficiency or anti-FGL1 mAb blockade restrained liver metastasis and tumor growth. The antitumor efficacy of FGL1 blockade depended on both CD8^+^ T and NK cells. This study reveals critical regulatory roles of FGL1 in the liver immune microenvironment and provides a new target of immune checkpoints for liver cancer immunotherapy.

## Results

### Hepatocyte-derived FGL1 is associated with the liver immune microenvironment.

**Figure 1:**
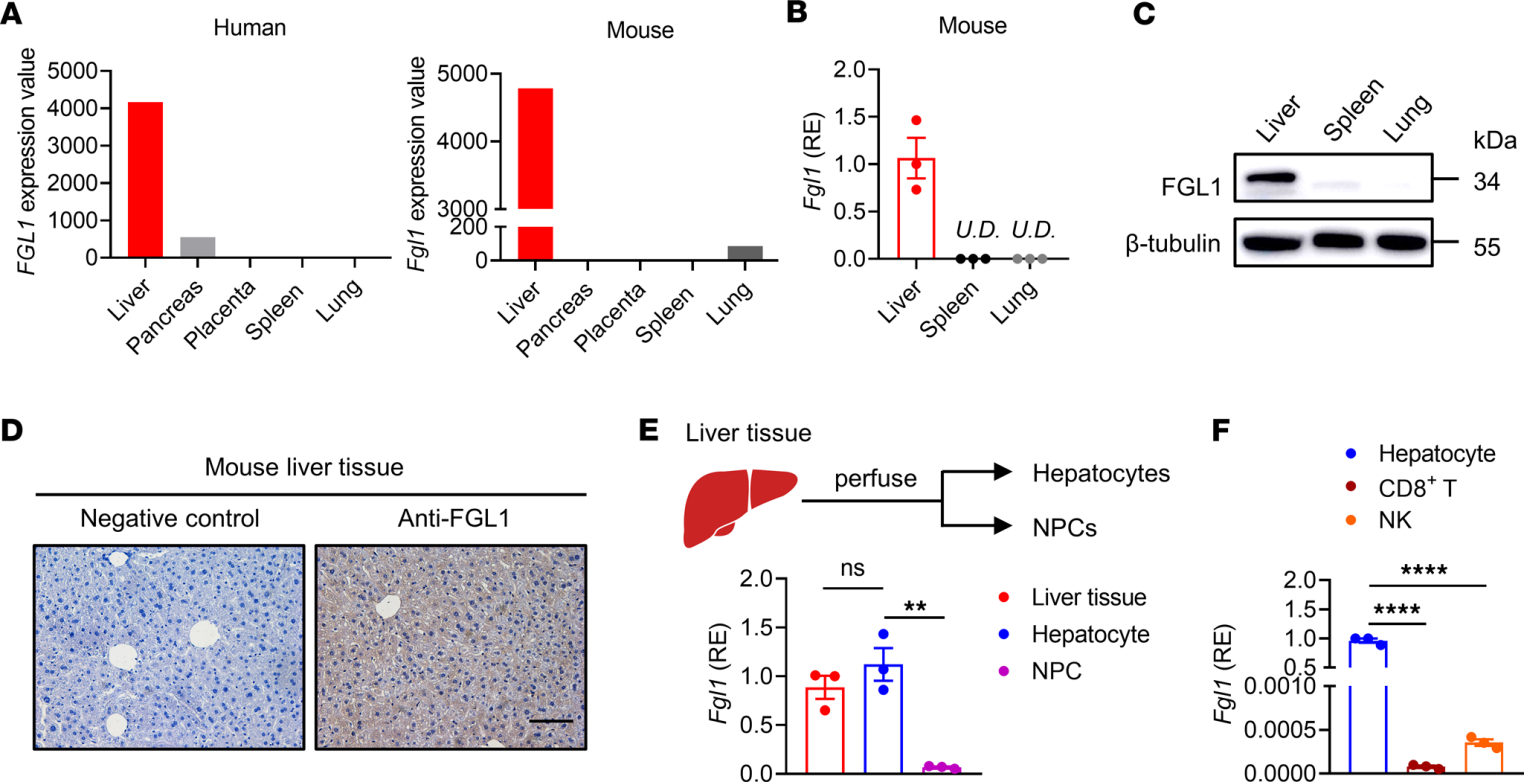
FGL1 is specifically expressed by hepatocytes. (**A**) *Fgl1* mRNA expression in human tissues (left) from the Genotype-Tissue Expression database and mouse tissues (right) from the BioGPS database. (**B** and **C**) Liver, spleen, and lung tissues of wild-type C57BL/6 (WT B6) mice were harvested to analyze FGL1 expression. (**B**) The mRNA expression levels of *Fgl1* in the liver, spleen, and lung. Relative expression (RE) levels are shown (*n* = 3/group). (**C**) The protein levels of FGL1 in the liver, spleen, and lung were detected by Western blot. β-Tubulin was the internal control. (**D**) Immunohistochemical staining of FGL1 protein in liver tissue. Bar, 100 μm. (**E**) The mRNA expression level of *Fgl1* in hepatocytes compared with nonparenchymal cells (NPCs). Hepatocytes and NPCs of WT mice were isolated (*n* = 3/group). (**F**) Liver mononuclear cells (MNCs) were isolated, and CD8^+^ T and NK cells were obtained by magnetic-activated cell sorting. The mRNA expression levels of *Fgl1* in hepatocytes (*n* = 3/group), CD8^+^ T and NK cells are shown. CD8^+^ T cells were sorted from the livers of 7 WT mice, and NK cells were sorted from the livers of 11 WT mice; each point represents a replicate (*n* = 3/group). Comparisons were performed by using 1-way ANOVA followed by Tukey’s multiple comparisons test (**E** and **F**). Data are presented as the mean ± SEM (**B**, **E**, and **F**). ****P < 0.0001, **P < 0.01. U.D., undetectable.

According to the database, FGL1 is expressed at high levels in the liver tissue of both humans and mice under normal physiological conditions (Figure 1A). To confirm this, *Fgl1* mRNA and protein expression levels were further detected in mouse liver compared with spleen and lung. Higher *Fgl1* mRNA (Figure 1B) and FGL1 protein (Figure 1C) levels were detected in the mouse liver but not in the spleen or lung. FGL1 was specifically expressed by hepatocytes but not nonparenchymal cells (NPCs) in the liver, as shown by positive staining by immunohistochemical analysis (Figure 1D), and a high relative expression level was detected in hepatocytes by quantitative PCR assay (Figure 1E), but not in NPCs, including liver-infiltrating lymphocytes such as hepatic CD8^+^ T and NK cells (Figure 1F). These data demonstrated that hepatocytes specifically and highly expressed FGL1 under normal physiological conditions.

**Figure 2:**
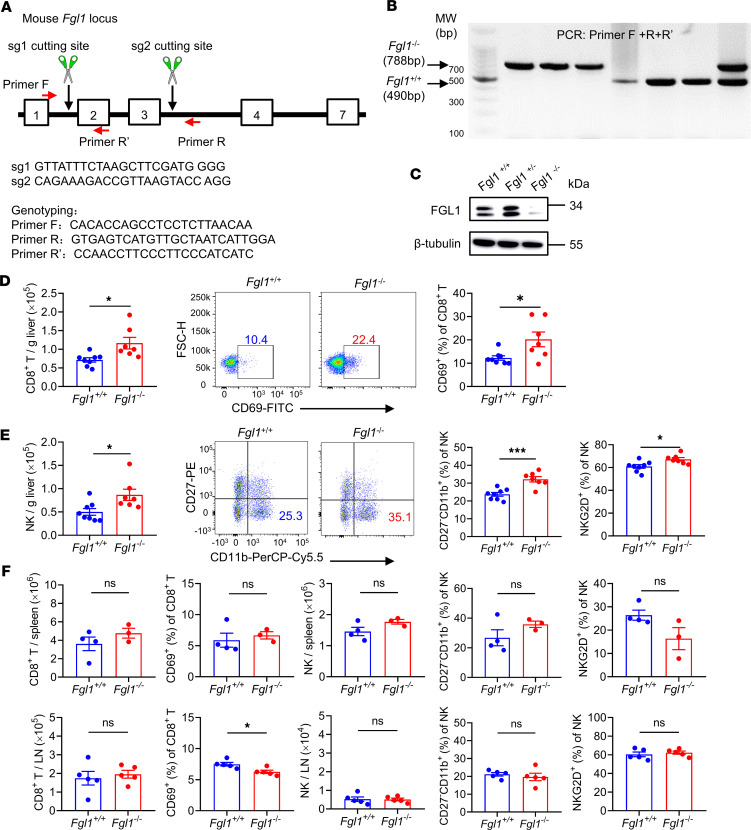
*Fgl1*-deficient mice exhibit accumulation and activation of CD8^+^ T and NK cells in the liver. (**A**) Generation and identification method of *Fgl1*-deficient mice. (**B**) Genotyping results for *Fgl1*^+/+^ and *Fgl1*^–/–^ alleles in mice. (**C**) Western blot analysis of FGL1 protein in the livers of *Fgl1*^+/+^, *Fgl1*^+/–^, and *Fgl1*^–/–^ mice. β-Tubulin was the internal control. MNCs from the livers, spleens, and lymph nodes (LN) of *Fgl1*^+/+^ and *Fgl1*^–/–^ mice were isolated and analyzed by flow cytometry. (**D**) Absolute numbers of hepatic CD8^+^ T cells (left). The expression of CD69 on hepatic CD8^+^ T cells is shown in a representative dot plot (middle). Frequencies of CD69 on hepatic CD8^+^ T cells (right). (*n* = 8 for *Fgl1*^+/+^ mice; *n* = 7 for *Fgl1*^–/–^ mice.) (**E**) Absolute numbers of hepatic NK cells (left). Percentages of the CD27^–^CD11b^+^ NK cell subset in the liver are shown by a representative dot plot and were statistically analyzed (middle). Frequencies of NKG2D on hepatic NK cells. (*n* = 8 for *Fgl1*^+/+^ mice; *n* = 7 for *Fgl1*^–/–^ mice.) (**F**) Absolute numbers of CD8^+^ T cells and frequencies of CD69 on CD8^+^ T cells in the spleen and LN of *Fgl1*^+/+^ and *Fgl1*^–/–^ mice. Absolute numbers of NK cells, percentages of the CD27^–^CD11b^+^ NK cell subset, and frequencies of NKG2D on NK cells in the spleen and LN of *Fgl1*^+/+^ and *Fgl1*^–/–^ mice. Spleen (*n* = 4 for *Fgl1*^+/+^ mice; *n* = 3 for *Fgl1*^–/–^ mice), LN (*n* = 5/group). Data are pooled from 2 independent experiments (**D** and **E**) and representative of at least 2 independent experiments (**F**). Comparisons were performed by using 2-tailed unpaired Student’s *t* test (**D**–**F**). Data are presented as the mean ± SEM (**D**–**F**). ***P < 0.001, *P < 0.05.

To explore the role of FGL1 in the liver immune microenvironment, *Fgl1*-deficient (*Fgl1*^–/–^) mice were generated by a CRISPR/Cas9-mediated genome-engineering strategy (Figure 2A) and identified by PCR and Western blot analysis (Figure 2, B and C). The absolute numbers of CD8^+^ T and NK cells were significantly increased in the livers of *Fgl1*^–/–^ mice (Figure 2, D and E), while the numbers of natural regulatory T cells (nTregs) and induced Tregs (iTregs) were decreased in the livers of *Fgl1*^–/–^ mice (Supplemental Figure 1, A–D; supplemental material available online with this article; https://doi.org/10.1172/jci.insight.173215DS1), and no differences in the number of plasmacytoid dendritic cells (pDCs) were detected (Supplemental Figure 1, E–H). Intrahepatic CD8^+^ T cells showed upregulated expression of the activation marker CD69 in *Fgl1*^–/–^ mice (Figure 2D), and intrahepatic NK cells became more mature, as shown by an increased percentage of the CD27^–^CD11b^+^ NK cell subset and a higher expression level of the activation receptor natural-killer group 2 member D (NKG2D) in *Fgl1*^–/–^ mice (Figure 2E). Interestingly, there were no significant differences in CD8^+^ T or NK cells in the spleen or lymph nodes between *Fgl1*^–/–^ mice and WT control mice (Figure 2F), indicating that hepatocyte-derived FGL1 is especially associated with the liver immune microenvironment. *Fgl1* deficiency had no obvious impact in the adult mice, since no significant alterations in the morphology, histology, and serum levels of pro-inflammatory cytokines including IL-1α, IL-6, IFN-γ, and TNF-α were observed (Supplemental Figure 2, A–F).

**Figure 3:**
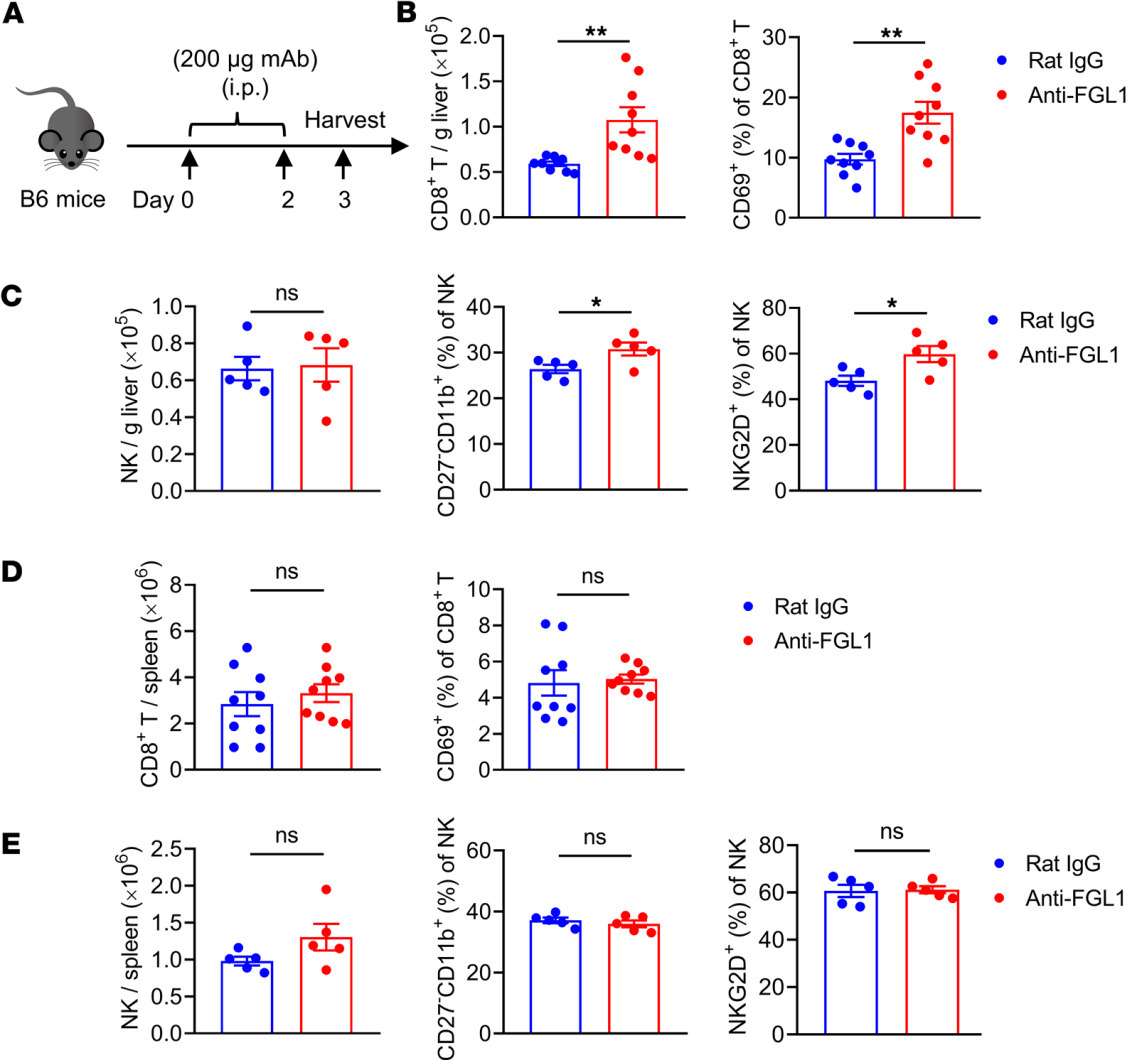
Anti-FGL1 mAb treatment promotes the accumulation and slight activation of CD8^+^ T and NK cells under physiological conditions. (**A**) Experimental protocol for anti-FGL1 mAb treatment used (**B**–**E**): WT mice were injected intraperitoneally (i.p.) with anti-FGL1 mAb (200 μg) or control rat IgG (200 μg) on day 0 and day 2, and then on day 3, livers and spleens were harvested for flow cytometry analysis of CD8^+^ T and NK cells. (**B**) Absolute numbers of hepatic CD8^+^ T cells (left) and frequencies of CD69 on hepatic CD8^+^ T cells (right) in the rat IgG group and anti-FGL1 group (*n* = 9/group). (**C**) Absolute numbers of hepatic NK cells in the rat IgG group and anti-FGL1 group (left). Percentages of the CD27^–^CD11b^+^ NK cell subset (middle) and frequencies of NKG2D (right) on hepatic NK cells in the rat IgG group and anti-FGL1 group (*n* = 5/group). (**D**) Absolute numbers of splenic CD8^+^ T cells (left) and frequencies of CD69 (right) on splenic CD8^+^ T cells in the rat IgG group and anti-FGL1 group (*n* = 9/group). (**E**) Absolute numbers of splenic NK cells (left). Frequencies of the CD27^–^CD11b^+^ NK cell subset (middle) and percentages of NKG2D (right) on splenic NK cells in the rat IgG group and anti-FGL1 group (*n* = 5/group). Data are pooled from 2 independent experiments (**B** and **D**) and representative of at least 2 independent experiments (**C** and **E**). Comparisons were performed by using 2-tailed unpaired Student’s *t* test (**B**–**E**). Data are presented as the mean ± SEM (**B**–**E**). **P < 0.01, *P < 0.05.

To further characterize the function of FGL1, we treated WT mice with a function-blocking monoclonal antibody (mAb) against FGL1 (anti-FGL1 mAb) (Figure 3A). Consistent with the findings observed in *Fgl1*^–/–^ mice, intrahepatic CD8^+^ T cells were significantly increased and showed higher expression of CD69 in anti-FGL1–treated mice compared with control IgG–treated mice (Figure 3B). Although there was no change in the number of intrahepatic NK cells, NK cells showed a significant increase in the percentage of the CD27^–^CD11b^+^ NK cell subset and expressed higher levels of the activation receptor NKG2D after anti-FGL1 mAb treatment (Figure 3C). Similarly, there were no significant differences in CD8^+^ T or NK cells in the spleen between anti-FGL1–treated mice and control IgG–treated mice (Figure 3, D and E), further verifying that FGL1 plays a critical regulatory role in the liver immune microenvironment.

### FGL1 deficiency inhibits liver metastasis of CRC.

**Figure 4:**
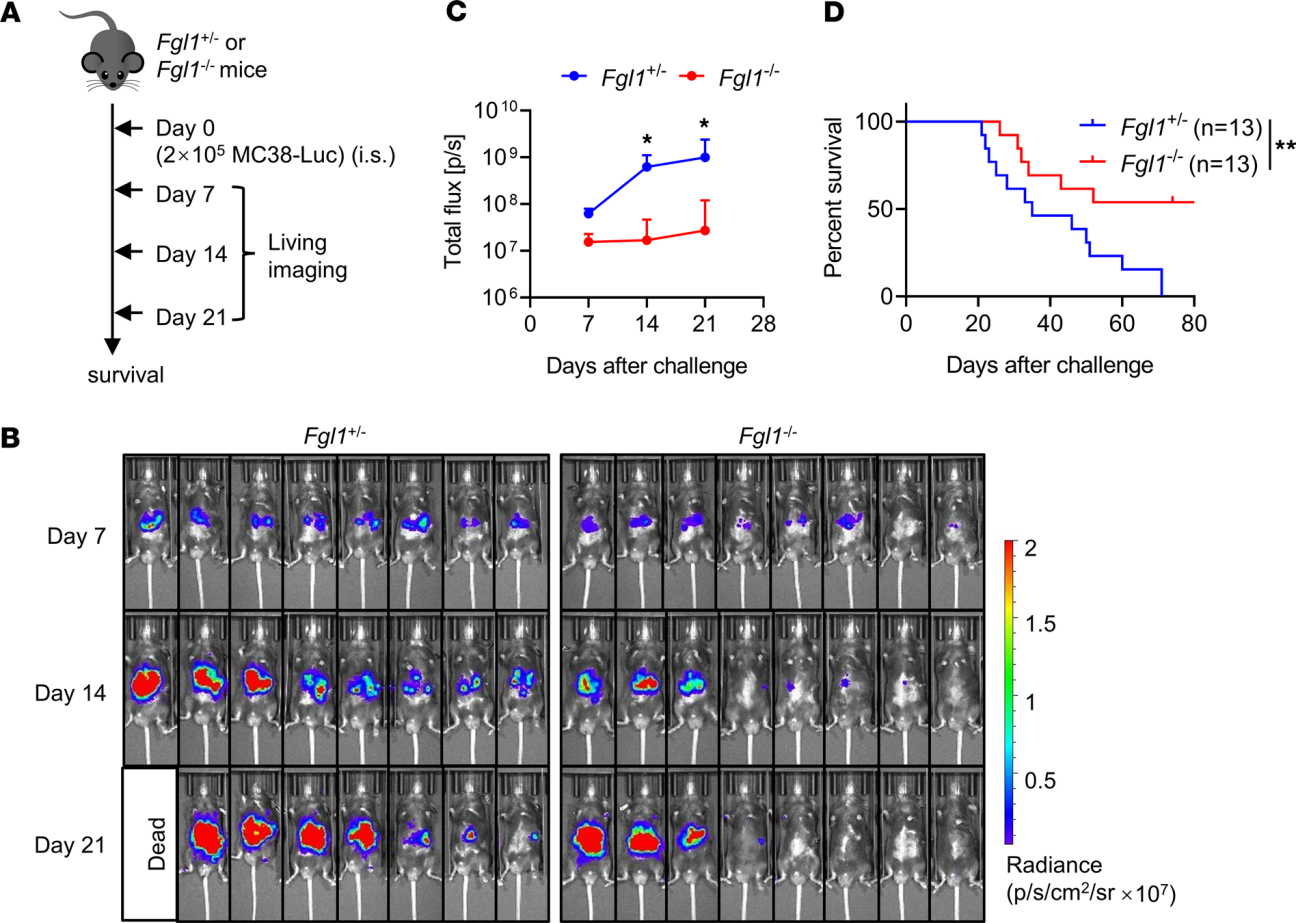
*Fgl1*-deficient mice display inhibited CRC liver metastasis and prolonged survival. (**A**) Experimental protocol for the CRC liver metastasis model used (**B**–**D**): *Fgl1*^+/–^ mice and *Fgl1*^–/–^ mice were injected intrasplenically (i.s.) with 2 × 10^5^ MC38-Luc tumor cells on day 0. (**B**) In vivo bioluminescence imaging of tumor metastases in mice at 7, 14, and 21 days after tumor challenge. There were 8 mice in each group. Data are presented as radiance values (p/s/cm^2^/sr), photons (p) per second per cubic centimeter of tissue that radiate into a solid angle of 1 steradian (sr). (**C**) Quantification of the radiance values. (**D**) Survival of mice challenged with MC38-Luc tumor cells. There were 13 mice in each group. Data are representative of 3 independent experiments (**B** and **C**) or pooled from 2 independent experiments (**D**). Comparisons were performed by using 2-way ANOVA followed by Holm-Šídák multiple comparisons test (**C**) and the log-rank (Mantel-Cox) test (**D**). Data are presented as the mean ± SEM (**C**). **P < 0.01, *P < 0.05.

The liver is a common site of tumor metastasis. CRC (30–50% of cases), pancreatic cancer (30%–40% of cases), cutaneous melanoma (10–20% of cases), and breast cancer (6%–38% of cases) metastasize to the liver (2). Here, an experimental model of CRC liver metastasis generated by the spleen transfer of MC38-Luc tumor cells was used to explore the role of FGL1 in the liver tumor microenvironment (Figure 4A). Significantly weaker radiance values were observed in the livers of *Fgl1^–/–^* mice compared with *Fgl1^+/–^* control mice at 14 and 21 days after MC38-Luc challenge by in vivo bioluminescence imaging (Figure 4, B and C), indicating that liver metastasis was significantly inhibited in *Fgl1^–/–^* mice. Furthermore, *Fgl1*^–*/*–^ mice exhibited significantly prolonged survival compared with *Fgl1^+/–^* control mice (Figure 4D). These results demonstrated that FGL1 deficiency significantly inhibited liver metastasis of CRC.

### FGL1 blockade restrains liver metastasis and tumor growth.

**Figure 5:**
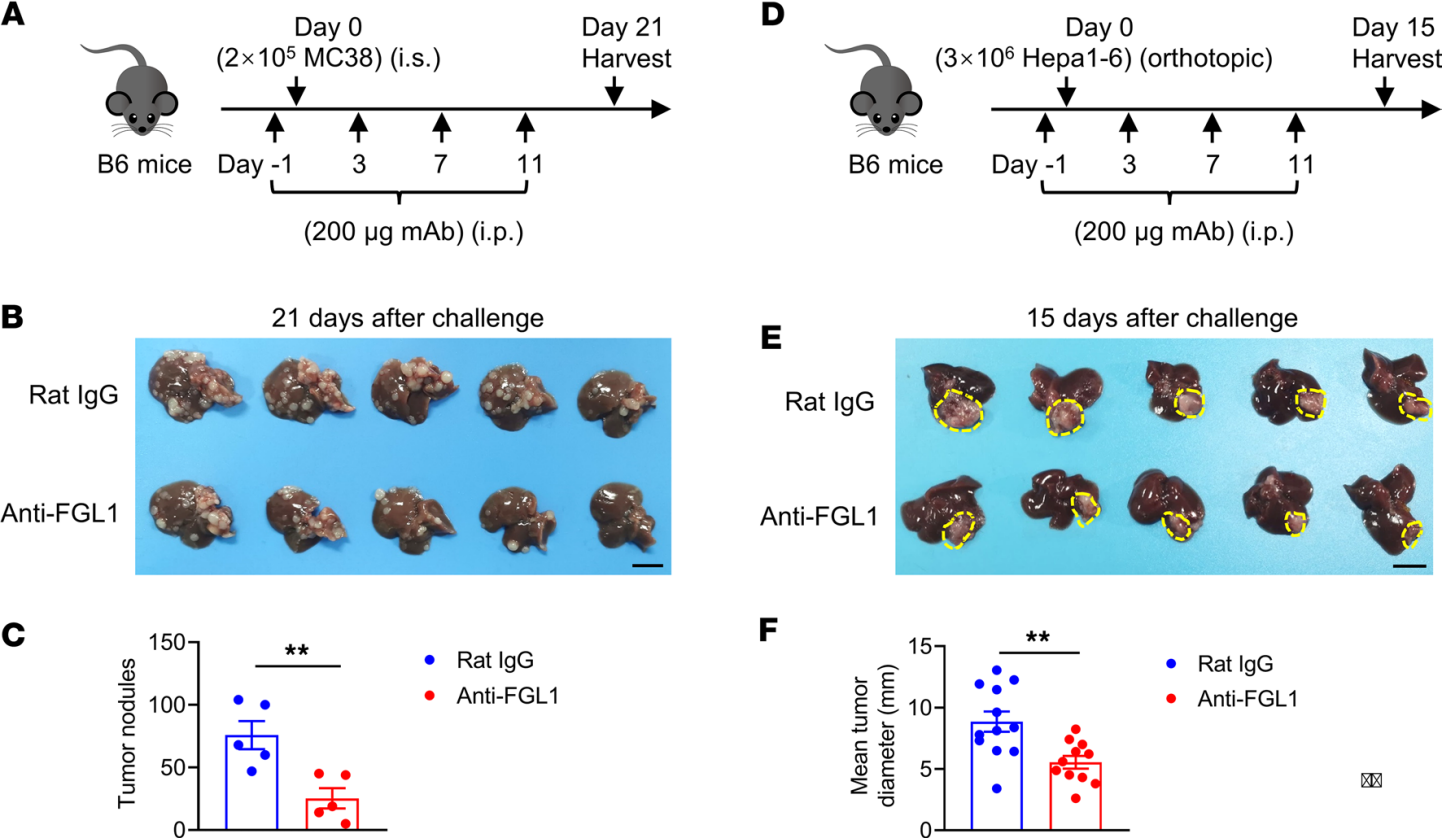
Pretreatment with the anti-FGL1 mAb slows the growth of liver metastatic and orthotopic tumors. (**A**) Experimental protocol for the CRC liver metastasis model used in **B** and **C**. WT mice were injected intraperitoneally (i.p.) with 200 μg anti-FGL1 mAb or control rat IgG once every 4 days 4 times, starting 1 day before tumor cell challenge. On day 0, 2 × 10^5^ MC38 tumor cells were intrasplenically (i.s.) challenged. On day 21 after challenge, the livers were harvested. (**B**) Representative photograph of livers. Bar, 1 cm. (**C**) Number of liver tumor nodules (*n* = 5/group). (**D**) Experimental protocol for the orthotopic liver cancer model used in **E** and **F**. WT mice were injected intraperitoneally (i.p.) with 200 μg anti-FGL1 mAb or control rat IgG once every 4 days 4 times, starting 1 day before tumor cell challenge. On day 0, 3 × 10^6^ Hepa1-6 tumor cells were orthotopically challenged. (**E**) Representative photograph of livers. Bar, 1 cm. (**F**) Mean tumor diameter (*n* = 12 for the rat IgG group; *n* = 11 for the anti-FGL1 group) 15 days after challenge. Data are representative of 2 independent experiments (**C**) and pooled from 2 independent experiments (**F**). Each symbol (**C** and **F**) represents an individual mouse. Comparisons were performed by using 2-tailed unpaired Student’s *t* test (**C** and **F**). Data are presented as the mean ± SEM (**C** and **F**). **P < 0.01.

FGL1 deficiency inhibited liver metastasis of CRC, indicating that a high level of FGL1 in the liver might be associated with liver cancer development and progression. First, WT mice were pretreated with anti-FGL1 mAb 1 day before tumor cell inoculation by using a CRC liver metastasis model of MC38 tumor cells and a liver orthotopic tumor model of Hepa1-6 tumor cells (Figure 5, A and D). On day 21 after challenge, the morphology of the liver showed that pretreatment with the anti-FGL1 mAb inhibited liver metastasis of the MC38 tumor cells (Figure 5B), which was further verified by the significantly decreased number of tumor nodules in the liver of the anti-FGL1 mAb–treated mice (Figure 5C). In the liver orthotopic tumor of Hepa1-6 in situ inoculation, the morphology of liver showed that pretreatment with the anti-FGL1 mAb inhibited orthotopic tumor growth (Figure 5E), which was further verified by the significantly decreased tumor diameter in the livers of the anti-FGL1 mAb–treated mice (Figure 5F). These results indicated that pretreatment with the anti-FGL1 mAb slowed the growth of liver metastatic and orthotopic tumors.

**Figure 6:**
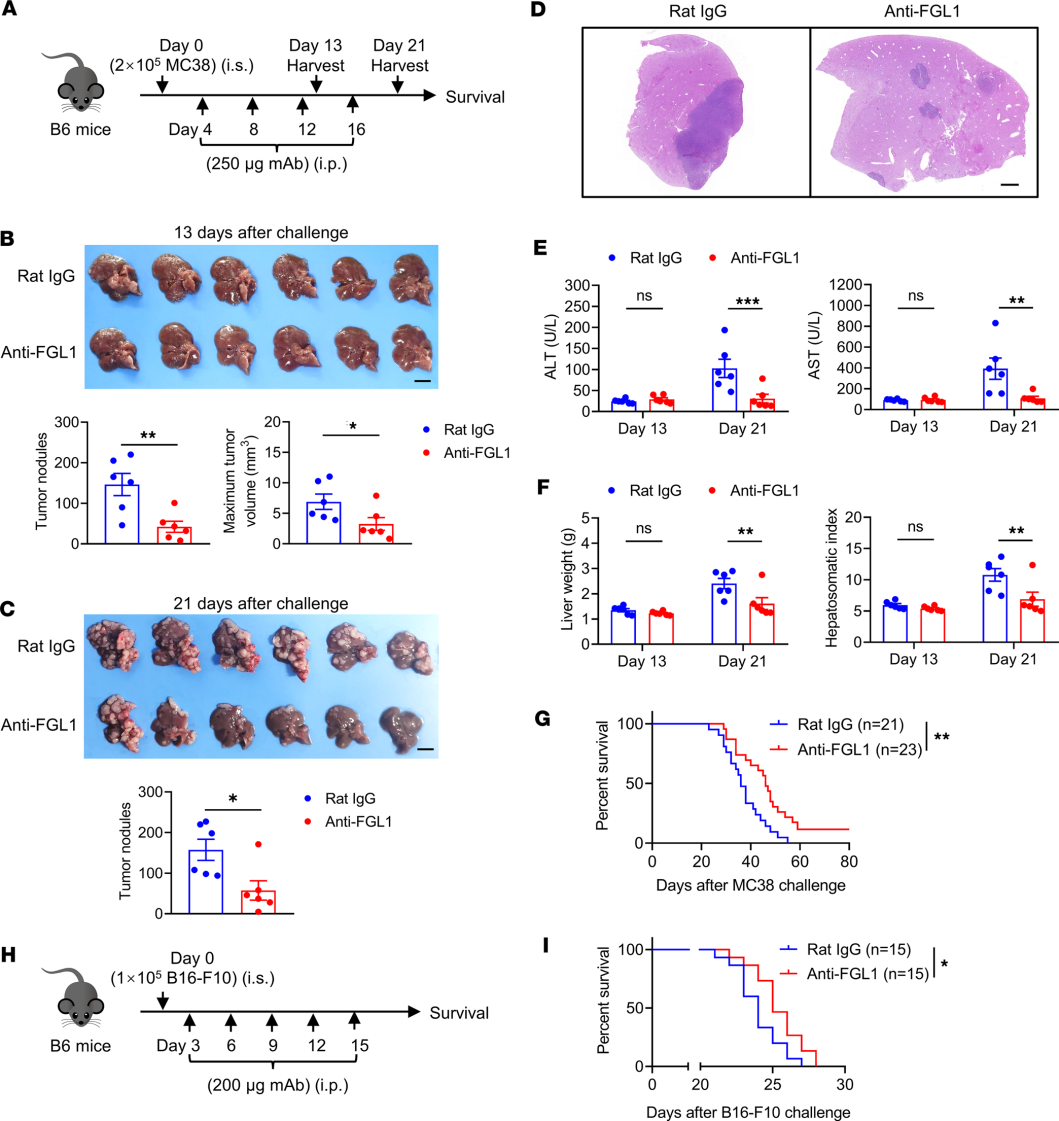
Blockade of FGL1 delays the progression of liver metastases. (**A**) Experimental protocol for the CRC liver metastasis model used (**B**–**G**): On day 0, WT mice were intrasplenically (i.s.) challenged with 2 × 10^5^ MC38 tumor cells and then treated intraperitoneally (i.p.) with 250 μg anti-FGL1 mAb or control rat IgG 4 times 4 days after tumor cell challenge (once every 4 days). (**B** and **C**) Representative photograph of livers, number of liver tumor nodules, and maximum tumor volumes at 13 and 21 days after challenge (*n* = 6/group). Bar, 1 cm. (**D**) Representative histopathological images of the liver tissues at 17 days after challenge. Bar, 1 mm. (**E**) Serum levels of ALT and AST and (**F**) liver weight and hepatosomatic index of mice at 13 days and 21 days after challenge (*n* = 6/group). (**G**) Survival of mice challenged with MC38 tumor cells (*n* = 21 for the rat IgG group; *n* = 23 for the anti-FGL1 group). (**H**) Experimental protocol for the melanoma liver metastasis model used in **I**: On day 0, WT mice were i.s. challenged with 1 × 10^5^ B16-F10 tumor cells and then treated i.p. with 200 μg anti-FGL1 mAb or control rat IgG 3 days after tumor cell challenge 5 times (once every 3 days). (**I**) Survival of mice challenged with B16-F10 tumor cells (*n* = 15/group). Data are representative of at least 2 independent experiments (**B** and **C**) and pooled from 3 (**G**) or 2 (**I**) independent experiments. Comparisons were performed by using 2-tailed unpaired Student’s *t* test (**B** and **C**), 2-way ANOVA followed by Holm-Šídák multiple comparisons test (**E** and **F**), and the log-rank (Mantel-Cox) test (**G** and **I**). Data are presented as the mean ± SEM (**B**, **C**, **E**, and **F**). ***P < 0.001, **P < 0.01, *P < 0.05.

Furthermore, by using an established CRC liver metastasis model of MC38 tumor cells, we explored the therapeutic effect of the anti-FGL1 mAb i.p. once every 4 days 4 times (Figure 6A). On day 13 after MC38 challenge, a significant reduction in tumor nodules and maximum tumor volumes in the liver was observed in anti-FGL1 mAb–treated mice compared with control IgG–treated mice (Figure 6B). Similarly, a significant reduction in tumor nodules in the liver was also observed in anti-FGL1 mAb–treated mice on day 21 after MC38 challenge (Figure 6C). Additionally, anti-FGL1 mAb treatment induced the reduction of MC38 tumors in the liver, shown by histological analysis (Figure 6D), decreased serum levels of alanine aminotransferase (ALT) and aspartate aminotransferase (AST) (Figure 6E), liver weight, and hepatosomatic index (Figure 6F), indicating the effects of FGL1 blockade on tumor elimination. In the CRC liver metastasis model, anti-FGL1 mAb–treated mice showed significantly prolonged survival after 4 treatments, with a median survival time ranging from 36 ± 1.37 days to 46 ± 1.60 days (Figure 6G). Additionally, the therapeutic effect of anti-FGL1 mAb i.p. once every 3 days 5 times was demonstrated by the significantly prolonged survival of mice with liver metastasis of B16-F10 tumor cells (Figure 6, H and I). Overall, FGL1 blockade with anti-FGL1 mAb inhibited liver metastasis and liver tumor growth.

### Both CD8^+^ T and NK cells are essential for the antitumor efficacy of FGL1 blockade.

Anti-FGL1 mAb treatment did not cause hepatocyte injury, as shown by histopathological images of liver tissues (Supplemental Figure 3A) and normal serum levels of ALT and AST (Supplemental Figure 3B), further indicating its role in promoting the antitumor activities of infiltrated lymphocytes in the liver.

**Figure 7:**
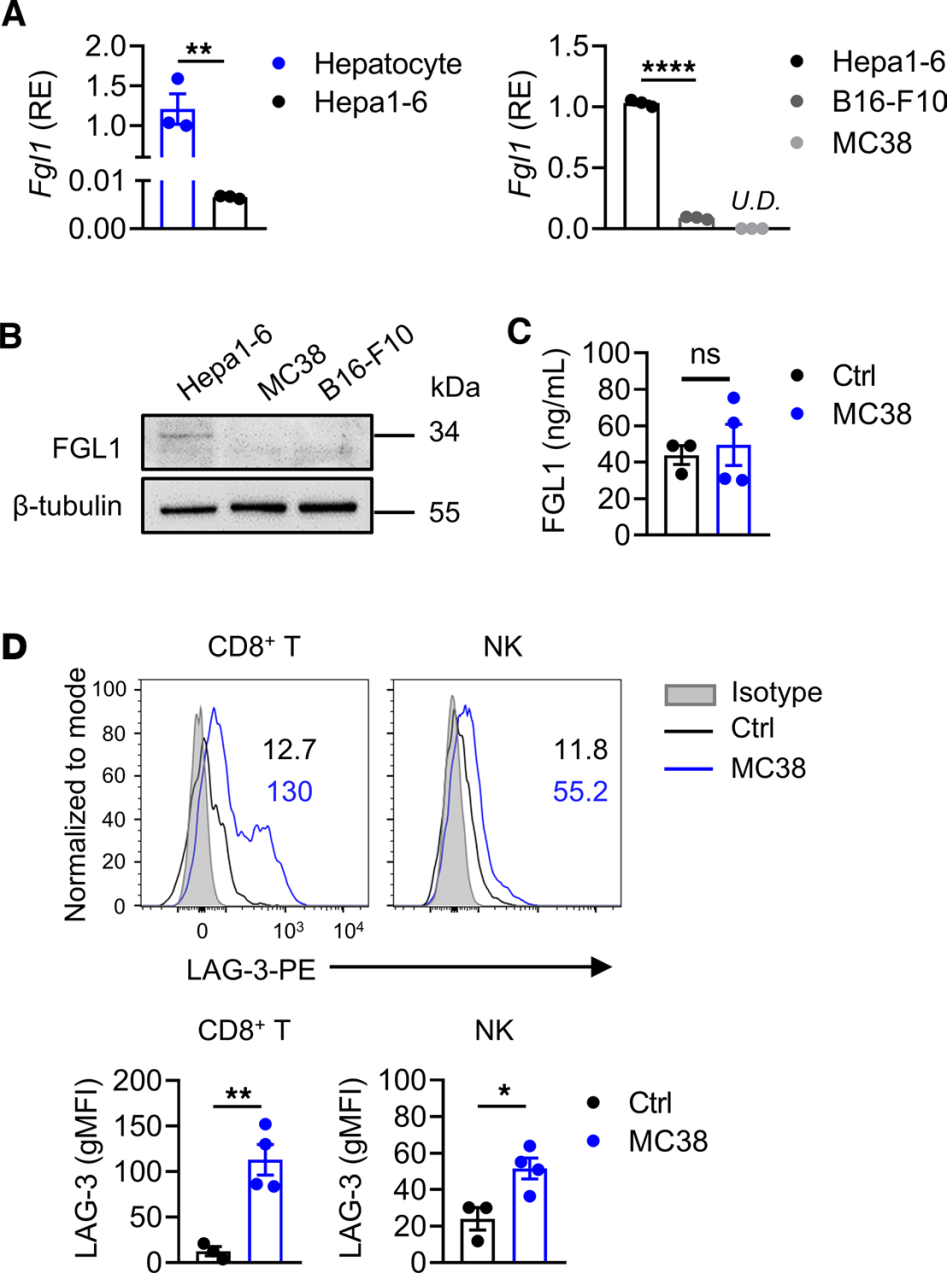
FGL1/LAG-3 interaction in the liver tumor microenvironment. (**A**) The mRNA expression levels of *Fgl1* in normal hepatocytes of WT mice and Hepa1-6 tumor cells (left) and in Hepa1-6 tumor cells compared with MC38 and B16-F10 tumor cells (right). Relative expression (RE) levels are shown (*n* = 3/group). (**B**) Western blot analysis of FGL1 protein in Hepa1-6, MC38, and B16-F10 tumor cells. β-Tubulin was the internal control. (**C**) The serum levels of FGL1 protein in MC38 tumor–bearing mice (*n* = 4) compared with control mice (*n* = 3). (**D**) Representative histograms (top) and geometric MFI (bottom) of LAG-3 expression on hepatic CD8^+^ T and NK cells from MC38-bearing mice (*n* = 4) compared with control mice (*n* = 3). Comparisons were performed by using 2-tailed unpaired Student’s *t* test (**A** left, **C** and **D**) and 1-way ANOVA followed by Tukey’s multiple comparisons test (**A** right). Data are presented as the mean ± SEM (**A**, **C**, and **D**). ****P < 0.0001, **P < 0.01, *P < 0.05. U.D., undetectable.

Compared with hepatocytes, tumor cells, including Hepa1-6, MC38, and B16-F10 cells, expressed lower levels of *Fgl1* mRNA (Figure 7A) and FGL1 protein (Figure 7B), indicating that hepatocytes are the major source of FGL1 in the liver tumor microenvironment. Notably, no significant differences in the serum levels of FGL1 protein were detected between the MC38 tumor–bearing mice and control mice (Figure 7C). However, the expression levels of its receptor LAG-3 were significantly increased on tumor-infiltrating hepatic CD8^+^ T and NK cells from MC38 tumor–bearing mice compared with control mice (Figure 7D), suggesting that there may be an FGL1/LAG-3 interaction in the liver tumor microenvironment.

**Figure 8:**
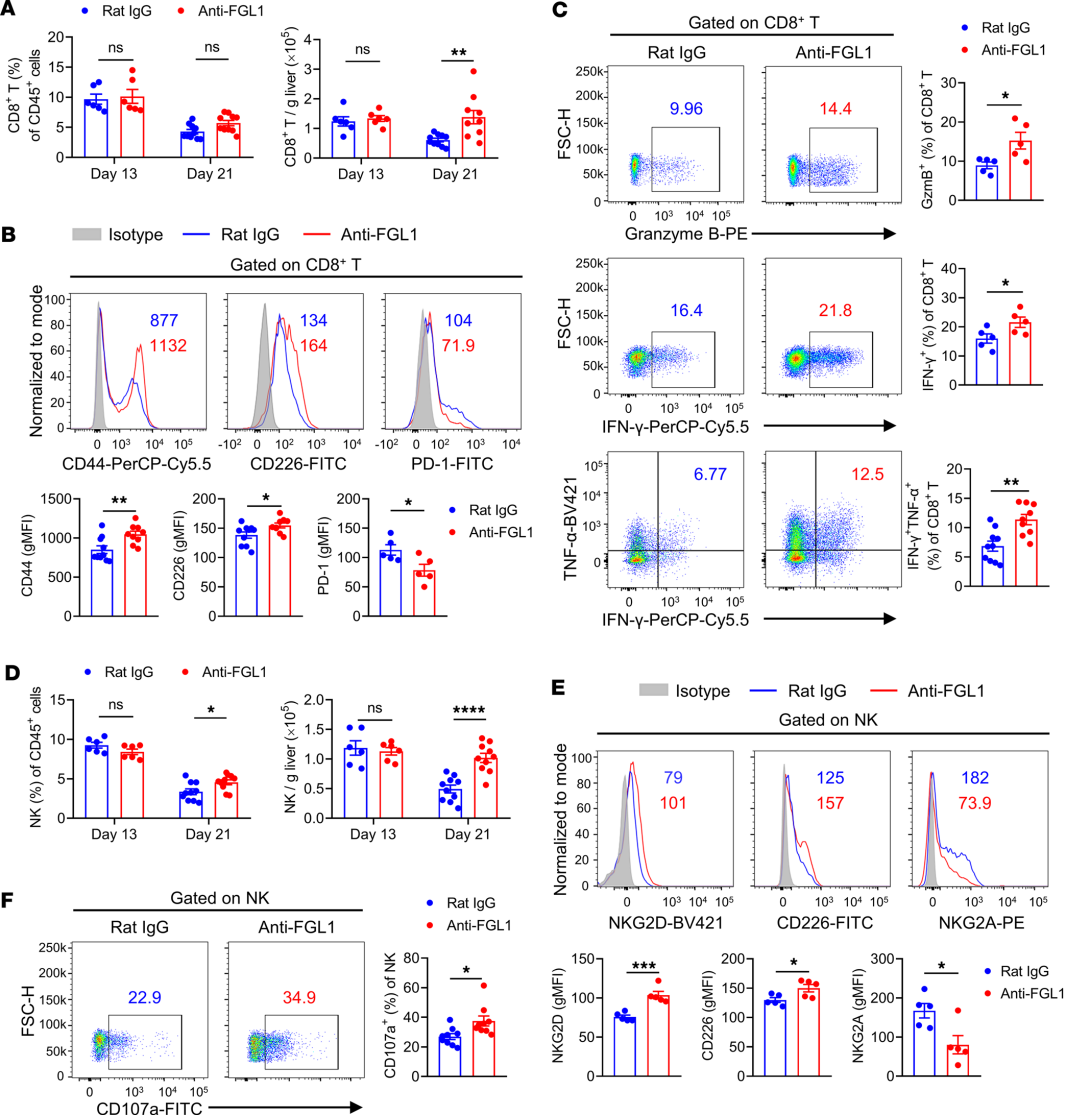
Blockade of FGL1 significantly promotes the infiltration and activation of CD8^+^ T and NK cells in the liver with tumors. On day 0, WT mice were intrasplenically (i.s.) challenged with 2 × 10^5^ MC38 tumor cells and then treated intraperitoneally (i.p.) with 250 μg anti-FGL1 mAb or control rat IgG 4 days after tumor cell challenge 4 times (once every 4 days), as described in Figure 6A. At 13 and 21 days after challenge, MNCs were isolated from the livers and analyzed by flow cytometry analysis. (**A**) Frequency and absolute numbers of CD8^+^ T cells in the liver (*n* = 6/group for day 13; *n* = 10/group for day 21). (**B**) Expression levels of CD44, CD226, and PD-1 on intrahepatic CD8^+^ T cells at 21 days after challenge. CD44, CD226 (*n* = 10 for the rat IgG group; *n* = 9 for the anti-FGL1 group), and PD-1 (*n* = 5/group). (**C**) Expression levels of granzyme B, IFN-γ, and TNF-α in intrahepatic CD8^+^ T cells at 21 days after challenge. Granzyme B, IFN-γ (*n* = 5/group), IFN-γ, and TNF-α (*n* = 10 for the rat IgG group; *n* = 9 for the anti-FGL1 group). (**D**) Frequency and absolute numbers of NK cells in the liver (*n* = 6/group for day 13; *n* = 10/group for day 21). (**E**) Expression levels of NKG2D, CD226, and NKG2A on intrahepatic NK cells at 21 days after challenge (*n* = 5/group). (**F**) Expression levels of CD107a on intrahepatic NK cells at 21 days after challenge (*n* = 9/group). Samples were compared using 2-tailed unpaired Student’s *t* test (**B**, **C**, **E**, and **F**) and 2-way ANOVA followed by Holm-Šídák multiple comparisons test (**A** and **D**). Data are presented as the mean ± SEM (**A**–**F**). ****P < 0.0001, ***P < 0.001, **P < 0.01, *P < 0.05.

Using an established CRC liver metastasis model of MC38 tumor cells, anti-FGL1 mAb treatment slightly (but not significantly) increased the frequency of CD8^+^ T cells and promoted the absolute number of CD8^+^ T cells in the liver on day 21 after MC38 challenge (Figure 8A). Furthermore, these intrahepatic CD8^+^ T cells exhibited upregulated expression of the activation markers CD44 and CD226 and downregulated expression of the inhibitory receptor PD-1 (Figure 8B) and exhibited increased expression of functional molecules, including granzyme B and IFN-γ (Figure 8C). In particular, the population of IFN-γ^+^TNF-α^+^CD8^+^ T cells was significantly increased in the livers of anti-FGL1 mAb–treated mice (Figure 8C). Additionally, anti-FGL1 mAb treatment increased the frequency of NK cells and the absolute number of NK cells in the liver on day 21 after MC38 challenge (Figure 8D). There was a significant increase in intrahepatic NK cell activation and cytotoxicity, as shown by increased expression of NKG2D and CD226, reduced expression of NKG2A (Figure 8E), and increased expression of CD107a (Figure 8F), in anti-FGL1 mAb–treated mice compared with control mice. Additionally, the frequency of nTregs was increased and the frequency of pDCs was decreased in the liver with MC38 tumor cells (Supplemental Figure 4, A and D); however, anti-FGL1 mAb treatment did not alter the frequencies or numbers of Tregs (Supplemental Figure 4, B and C) and pDCs (Supplemental Figure 4, E and F) in the liver.

**Figure 9:**
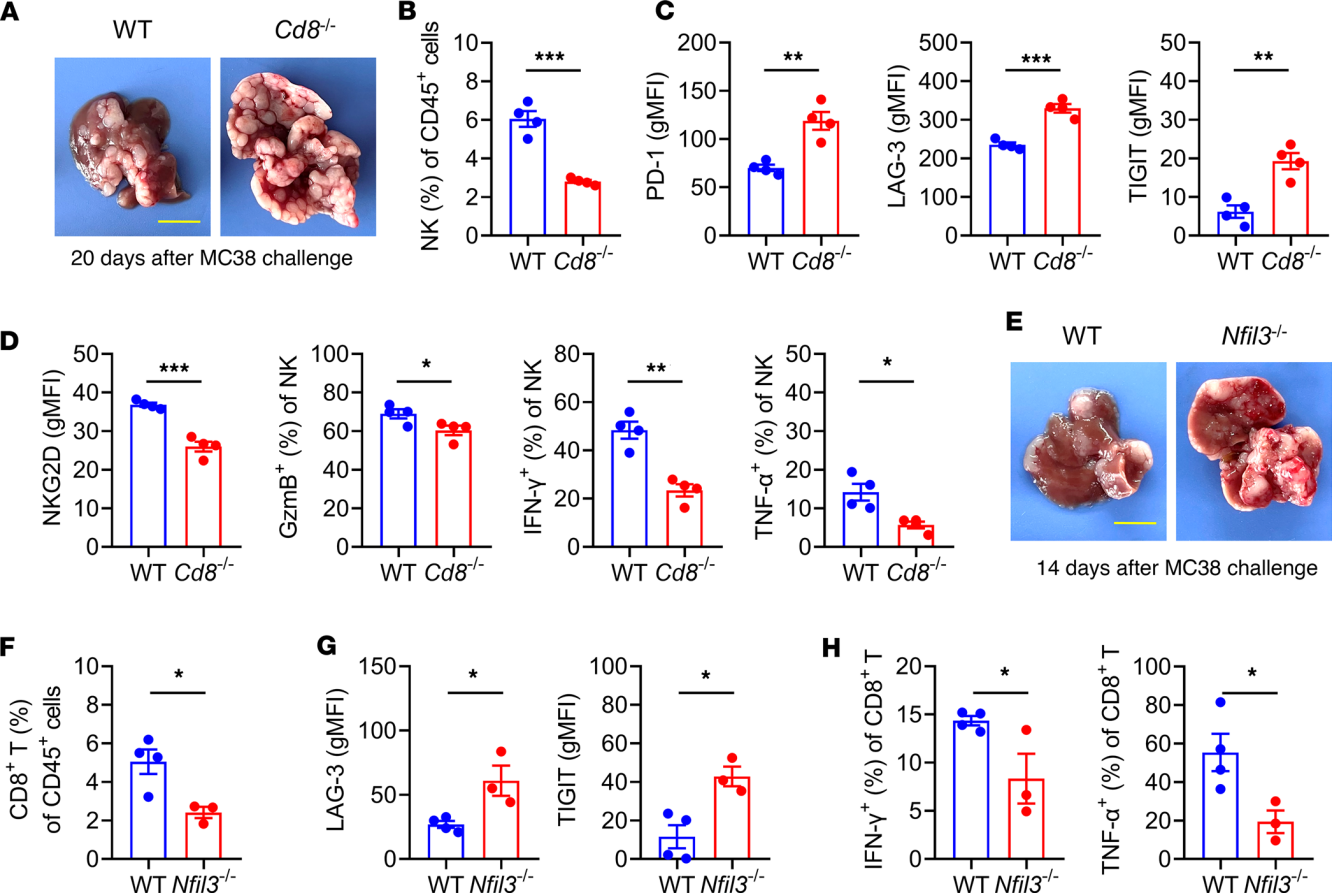
Both CD8^+^ T and NK cells play important roles and regulate reciprocally in the MC38 liver metastasis model. WT or *Cd8*^–/–^ mice were intrasplenically (i.s.) challenged with 2 × 10^5^ MC38 tumor cells. On day 20 after challenge, MNCs were isolated from the liver and analyzed by flow cytometry. (**A**) Representative photograph of livers. Bar, 1 cm. (**B**) Frequency of NK cells in the liver (*n* = 4/group). (**C**) Expression levels of PD-1, LAG-3, and TIGIT on intrahepatic NK cells (*n* = 4/group). (**D**) Expression levels of NKG2D, granzyme B, IFN-γ, and TNF-α on intrahepatic NK cells (*n* = 4/group). WT or *Nfil3^–/–^* mice (female) were i.s. challenged with 2 × 10^5^ MC38 tumor cells. On day 14 after challenge, MNCs were isolated from the liver and analyzed by flow cytometry. (**E**) Representative photograph of livers. Bar, 1 cm. (**F**) Frequency of CD8^+^ T cells in the liver. (**G**) Expression levels of LAG-3 and TIGIT on intrahepatic CD8^+^ T cells. (**H**) Expression levels of IFN-γ and TNF-α in intrahepatic CD8^+^ T cells. There were 4 mice in the WT group and 3 mice in the *Nfil3^–/–^* group, respectively. Comparisons were performed by using 2-tailed unpaired Student’s *t* test (**B**–**D** and **F**–**H**). Data are presented as the mean ± SEM. ***P < 0.001, **P < 0.01, *P < 0.05.

To elucidate the roles of CD8^+^ T and NK cells, *Cd8*^–/–^ mice and *Nfil3*^–/–^ mice were studied by using a CRC liver metastasis model of MC38 tumor cells. The deficiency of CD8^+^ T cells led to more liver metastasis (Figure 9A), along with a lower frequency of NK cells (Figure 9B), and NK cells expressed higher levels of PD-1, LAG-3, and TIGIT (Figure 9C) and lower levels of NKG2D, granzyme B, IFN-γ, and TNF-α (Figure 9D). On the other hand, the deficiency of NK cells also led to more liver metastasis (Figure 9E), along with a lower frequency of CD8^+^ T cells (Figure 9F), and CD8^+^ T cells expressed higher levels of LAG-3 and TIGIT (Figure 9G) and lower levels of IFN-γ and TNF-α (Figure 9H). These findings indicated that both CD8^+^ T and NK cells were involved in the control of MC38 liver metastasis, and CD8^+^ T cell deficiency impaired NK cell functions; meanwhile, NK cell deficiency destroyed CD8^+^ T cell functions, indicating the reciprocal regulation between NK cells and CD8^+^ T cells.

**Figure 10:**
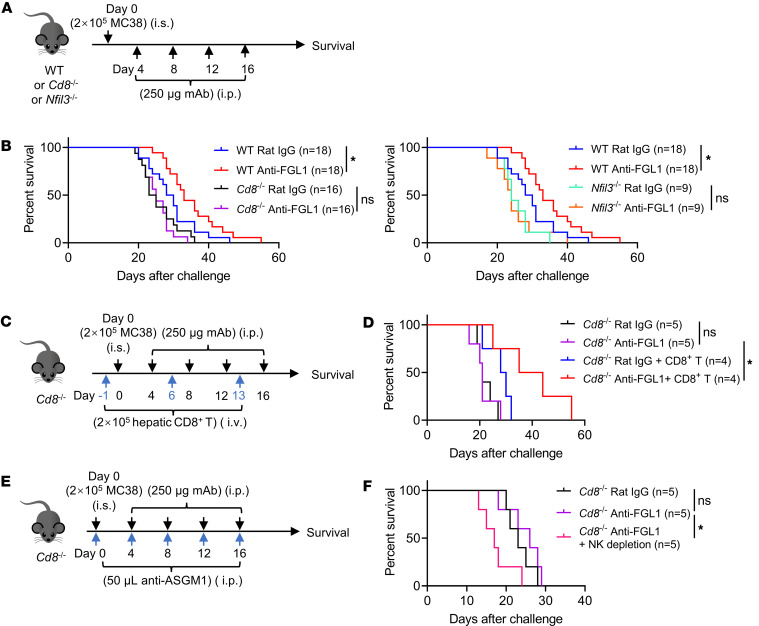
The antitumor efficacy of FGL1 blockade is dependent on CD8^+^ T and NK cells. (**A**) Experimental protocol for the CRC liver metastasis model used in **B**: On day 0, WT, *Cd8*^–/–^, or *Nfil3*^–/–^ mice were intrasplenically (i.s.) challenged with 2 × 10^5^ MC38 tumor cells and then treated intraperitoneally (i.p.) with 250 μg anti-FGL1 mAb or control rat IgG 4 days after tumor cell challenge 4 times (once every 4 days). (**B**) Survival of mice challenged with MC38 tumor cells (*n* = 18/group for the WT rat IgG group and WT anti-FGL1 group; *n* = 16/group for the *Cd8*^–/–^ rat IgG group and *Cd8*^–/–^ anti-FGL1 group; *n* = 9/group for the *Nfil3*^–/–^ rat IgG group and *Nfil3*^–/–^ anti-FGL1 group). Data are pooled from 3 independent experiments. (**C**) CRC liver metastasis model used as described in **A**: *Cd8*^–/–^ mice were intravenously (i.v.) transferred with 2 × 10^5^ hepatic CD8^+^ T cells from WT mice 3 times (once a week), starting 1 day before tumor cell challenge. (**D**) Survival of mice challenged with MC38 tumor cells (*n* = 4 or 5/group). (**E**) CRC liver metastasis model used as described in **A**: To deplete NK cells, mice were treated i.p. with 50 μL anti–asialo GM1 (anti-ASGM1) on day 0 (5 times once every 4 days). (**F**) Survival of mice challenged with MC38 tumor cells (*n* = 5/group). Comparisons were performed by using the log-rank (Mantel-Cox) test (**B**, **D**, and **F**). *P < 0.05.

Furthermore, anti-FGL1 mAb treatment was performed in *Cd8*^–/–^ mice and *Nfil3*^–/–^ mice challenged with MC38 tumor cells (Figure 10A). Anti-FGL1 mAb treatment prolonged the survival of WT mice with liver metastasis of CRC; however, no significant differences in survival were observed between anti-FGL1 mAb–treated *Cd8*^–/–^ mice and control IgG–treated *Cd8*^–/–^ mice (left, Figure 10B) or between anti-FGL1 mAb–treated *Nfil3*^–/–^ mice and control IgG–treated *Nfil3*^–/–^ mice (right, Figure 10B), demonstrating that the antitumor efficacy of FGL1 blockade was abrogated in *Cd8*^–/–^ mice or *Nfil3*^–/–^ mice because of deficiency of CD8^+^ T cells or NK cells, respectively. Adoptive transfer of CD8^+^ T cells restored antitumor immunity and prolonged the survival of *Cd8*^–/–^ mice treated with the anti-FGL1 mAb (Figure 10, C and D). NK cell depletion in *Cd8*^–/–^ mice resulted in the shortest survival time in tumor-bearing mice when treated with the anti-FGL1 mAb (Figure 10, E and F), further indicating the critical antitumor roles of NK cells in liver metastasis and their dependence on CD8^+^ T cells to show the therapeutic effects of anti-FGL1 antibody blockade. Overall, these results verified that both NK cells and cytotoxic T lymphocytes contributed to the therapeutic outcome of FGL1 blockade. NK cells alone were insufficient to mount effective antitumor immunity of FGL1 blockade in the absence of CD8^+^ T cells. CD8^+^ T cells, at least in part, played a critical role in supporting NK cells.

**Figure 11:**
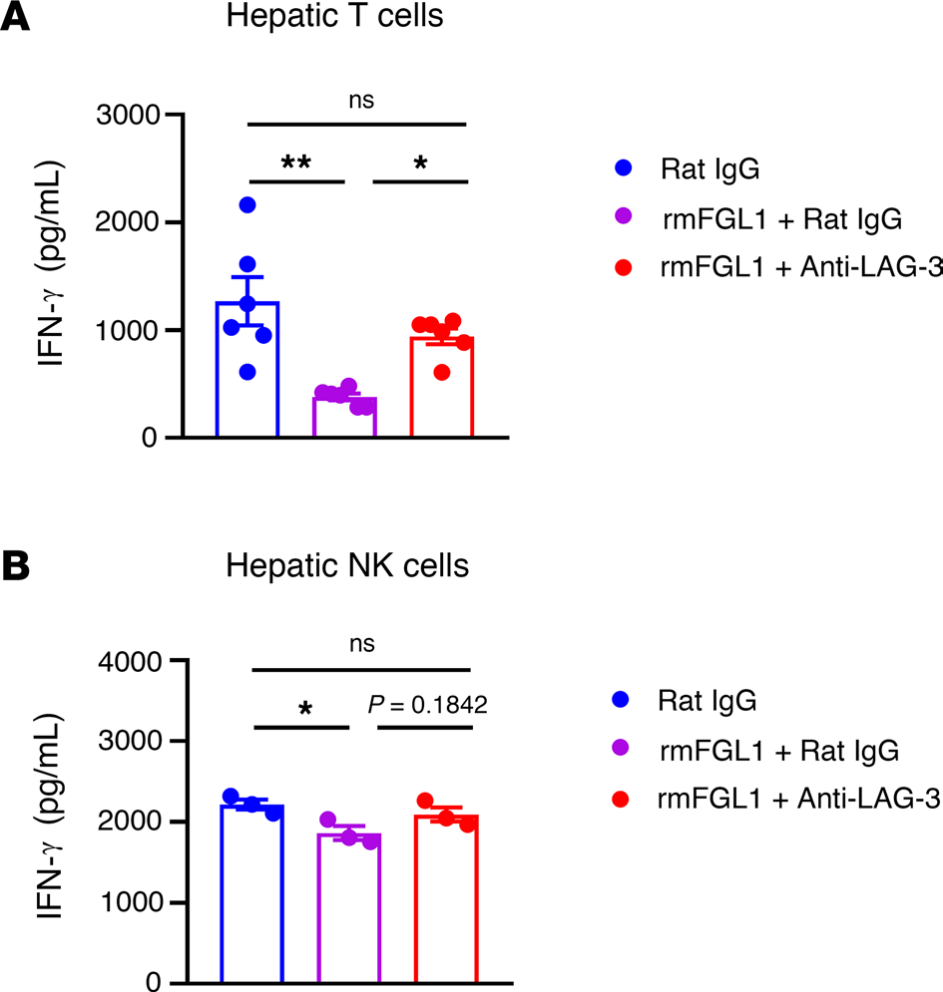
Recombinant mouse FGL1 protein inhibits hepatic T and NK cell function. (**A**) MNCs were isolated from mouse livers (*n* = 6/group) and activated by immobilized anti-CD3 mAb (2.5 μg/mL) and anti-CD28 (1.25 μg/mL). (**B**) Hepatic NK cells from 20 mice were sorted and then stimulated with IL-12 (10 ng/mL), IL-15 (50 ng/mL), and IL-18 (10 ng/mL). Each point represents a replicate (*n* = 3/group). Cells were cultured in the presence of recombinant mouse FGL1 (50 ng/mL), anti–LAG-3 mAb (1 μg/mL), or rat IgG (1 μg/mL) for 3 days. IFN-γ levels in the supernatant are shown. Data are representative of 2 independent experiments. Comparisons were performed by using 1-way ANOVA followed by Tukey’s multiple comparisons test (**A** and **B**). Data are presented as the mean ± SEM. **P < 0.01, *P < 0.05.

In vitro experiments demonstrated that recombinant mouse FGL1 significantly inhibited hepatic T cell function, as shown by the decreased levels of IFN-γ, and this suppression was diminished by treatment with the anti–LAG-3 mAb, indicating that FGL1 inhibited T cell function via its receptor LAG-3 (Figure 11A). Similarly, recombinant mouse FGL1 protein significantly inhibited NK cell function; however, anti–LAG-3 mAb treatment only partly reversed NK cell function, suggesting that FGL1 might inhibit NK cell function by some way in addition to LAG-3 (Figure 11B). In addition, recombinant mouse FGL1 protein had no direct effect on the proliferation (Supplemental Figure 5A) or migration (Supplemental Figure 5B) of MC38, B16-F10, and Hepa1-6 tumor cells in vitro, suggesting that the FGL1/LAG-3 interaction did not play a direct role in tumor metastasis or growth in our settings and further indicating the critical roles of CD8^+^ T and NK cells in the antitumor efficacy of FGL1 blockade.

## Discussion

In this study, the critical regulatory role of hepatocyte-derived FGL1 in the regional immune microenvironment of the liver was demonstrated. *Fgl1* deficiency or anti-FGL1 mAb blockade restrained liver metastasis and slowed the growth of orthotopic tumors, significantly prolonging the survival of tumor-bearing mice. FGL1 inhibited CD8^+^ T and NK cell functions via its receptor LAG-3, explaining to some extent why the liver has high susceptibility to primary tumors and is a common site of tumor metastasis and providing a strategy of checkpoint targeting for liver cancer immunotherapy.

Due to its specific anatomy and physiological functions, the liver is characterized by immune tolerance, demonstrated by hyposensitivity to intestine-derived antigens by the portal vein, recipients of liver allografts, protection of other organ grafts from the same donor, and chronic hepatotropic virus infection, such as HBV infection (25). Moreover, liver metastases disturb peripheral immune responses and then induce immune tolerance by macrophage-mediated elimination of CD8^+^ T cells (26). These immune cells that are predominantly enriched in the liver, such as Kupffer cells, conventional natural killer cells, type I innate lymphoid cells, and natural killer T cells, have been demonstrated to maintain the liver to be immune hyporesponsive, which may be related to its susceptibility to tumor development (27–30). Hepatocytes, two-thirds of the total cells in the liver, express PD-L1 and human leukocyte antigen-E (HLA-E) and secrete various kinds of immunoregulatory molecules, such as TGF-β, IL-10, and galectin-9, which are also involved in the induction of liver tolerance (31–35).

In this study, we found that hepatocytes specifically and highly expressed FGL1 under normal physiological conditions, and FGL1 played critical immunosuppressive roles in CD8^+^ T and NK cells in the liver at steady state, which was beneficial for maintaining immune tolerance of the liver (Figures 1–3). Thus, immune surveillance of CD8^+^ T and NK cells cannot be fully performed when malignant transformation occurs in the liver. On the other hand, tumor cells bring immunosuppressive microenvironments in the liver, including the recruitment of myeloid-derived suppressor cells (MDSCs) and Tregs and the induction of tumor-associated macrophages (36). Mutated hepatocytes upregulate many immunoregulatory signals, such as PD-L1, CD155, and HLA-E, and then immune cells are constantly stimulated and upregulate the expression of immune checkpoint receptors such as PD-1, TIGIT, CD96, and NKG2A, which are related to the exhaustion of T and NK cells (5, 37–39). In this study, increased expression levels of LAG-3 were detected on tumor-infiltrating hepatic CD8^+^ T and NK cells in MC38 tumor–bearing mice (Figure 7). The dual tolerant environment in the liver tends to make tumor cells grow uncontrollably, accounting for the high susceptibility to primary tumors and tumor metastasis.

Blockade of the FGL1/LAG-3 interaction by anti-FGL1 mAb or anti–LAG-3 mAb enhances antitumor immunity and shows therapeutic effects on an established mouse subcutaneous tumor model of the MC38 cell line (6). In this study, we further explored its critical role as a novel immune checkpoint in the progression of liver cancer since FGL1 is a hepatocyte-secreted protein. Genetic ablation or antibody blockade of FGL1 signaling inhibited liver metastasis and tumor growth by promoting CD8^+^ T and NK cell functions, suggesting that FGL1 is an immunotherapy target for liver cancer (Figures 4–6 and 8). Consistently, increased FGL1 expression in HCC has been shown to suppress CD8^+^ tissue-resident memory T cells and may result in CD8^+^ tissue-resident memory T cell exhaustion (20). Significantly elevated serum FGL1 was observed in patients with early-stage HBV-HCC (19). In this study, we demonstrated that hepatocytes were the major source of FGL1 in the liver tumor microenvironment, since tumor cells, including Hepa1-6, MC38, and B16-F10 cells, expressed significantly lower levels of FGL1 than hepatocytes (Figure 7). Both CD8^+^ T and NK cells are important for the control of liver metastasis (Figure 9) and are necessary for the therapeutic effect of FGL1 blockade. Neither CD8^+^ T cells nor NK cells alone optimize the therapeutic effect of FGL1 blockade (Figure 10). FGL1 inhibited hepatic T and NK cell function directly (Figure 11). No direct effects of FGL1 on tumor cell proliferation or migration were observed in vitro (Supplemental Figure 5). Thus, FGL1 blockade in liver tissues primarily affects the tumor microenvironment through the immunological regulation of CD8^+^ T and NK cells, which is a unique feature of liver tissue and may become an important means of inhibiting liver cancer.

Therapeutics targeting CTLA-4 or PD-1/PD-L1 have achieved obvious outcomes in advanced HCC, but they are limited when used in combination because of immune-related adverse events, such as hepatitis (40). In this study, anti-FGL1 mAb treatment did not cause hepatocyte injury in mice (Supplemental Figure 3); however, more preclinical trials are needed to prove the safety of FGL1 blockade.

The expression levels of hepatic FGL1 were significantly elevated in the condition of acute liver injury, such as partial hepatectomy and radiation-induced liver injury (25, 41), and in the extrahepatic induction of inflammation, such as subcutaneous injection of a known enhancer of IL-6, turpentine oil, and rheumatoid arthritis (42, 43). FGL1 protein was elevated in human HCC tissues since it was deacetylated and stabilized by Sirtuin 2, which was related to a poor prognosis (23). FGL1 is also produced by other human cancer cells, including lung cancer, prostate cancer, melanoma, CRC, breast cancer, brain tumor, gastric cancer, and clear cell renal cell carcinoma (6, 44, 45). Upregulated FGL1 in tumor tissues correlated with poor prognosis in patients with non–small cell lung cancer, metastatic melanoma, gastric cancer, or renal cell carcinoma (RCC) (6, 44, 45). A dual-targeting vaccine of FGL1 and carbonic anhydrase IX activated dendritic cell–mediated multifunctional CD8^+^ T cell antitumor immunity in advanced RCC (46). Dual immunological and proliferative regulation of FGL1 was observed in lung adenocarcinoma (LUAD). FGL1 may promote the secretion of IL-2, inducing the apoptosis of T cells, and promote the proliferation of LUAD cells via the Yin Yang 1/FGL1/myosin heavy chain 9 axis (47). FGL1 blockade may have dual effects on the development of certain cancers.

There are some remaining issues to be solved after this study. First, LAG-3 is considered a major receptor of FGL1. A recent study revealed that a recombinant protein of FGL1 fused with the pentameric domain of cartilage oligomeric matrix protein strongly bound to LAG-3–deficient T cells, suggesting the presence of FGL1-binding molecule(s) other than LAG-3 on activated T cells (48). In vitro experiments showed that the binding of LAG-3 to stable peptide-MHC class II complexes (pMHCII) but not to FGL1 induced T cell suppression, and LAG-3 mutants lacking FGL1-binding capacity retained suppressive activity, unlike those lacking stable pMHCII-binding capacity (48). Here, FGL1 suppressed T cell function, which could be completely reversed by anti–LAG-3 blockade, indicating the dependence on LAG-3 (Figure 11A). However, the anti–LAG-3 mAb could only partly reverse the inhibitory effect of FGL1 on NK cells (Figure 11B), and the underlying mechanisms deserve further investigation. Second, in addition to CD8^+^ T and NK cells, Tregs in the liver tumor microenvironment also express high levels of LAG-3. FGL1 is positively correlated with Treg and MDSC populations, as determined by analysis of the TIMER database (8), suggesting the regulatory effect of FGL1/LAG-3 signaling on immunosuppressive cells in the tumor microenvironment. In this study, anti-FGL1 mAb treatment slightly affected the frequency and number of Tregs and pDCs (Supplemental Figure 4). Whether anti-FGL1 mAb treatment affects the functions of Tregs and pDCs should be explored in the future. Third, our previous study demonstrated that TIGIT blockade, but not PD-L1 blockade, elicited potent antitumor immunity in a mouse model of naturally occurring HBV-related HCC, suggesting that TIGIT may be a novel target for HCC immunotherapy (5). Additionally, liver metastasis disturbs systemic antitumor immunity and causes immunotherapy resistance to PD-L1 blockade (26). It would be worthwhile to investigate the combined blockade of both PD-1/PD-L1 and FGL1/LAG-3 or CD155/TIGIT and FGL1/LAG-3 axes in further studies.

In conclusion, this study demonstrated that hepatocyte-derived FGL1 played critical regulatory roles in the liver immune microenvironment and accounted for liver metastasis and tumor growth by inhibiting CD8^+^ T and NK cell functions via the receptor LAG-3, providing a new strategy for liver cancer immunotherapy.

## Methods

### Sex as a biological variable.

Male and female mice were involved in this study. Sex was not considered as a biological variable.

### Mice.

Wild-type C57BL/6 (WT B6) mice were purchased from the Shanghai Experimental Animal Center (Shanghai, China) and GemPharmatech (Nanjing, China). C57BL/6 *Fgl1*^+/–^ and *Fgl1*^–/–^ mice were generated by the Laboratory Animal Center of University of Science and Technology of China (USTC) (Hefei, China) and bred in-house. C57BL/6 *Nfil3*^+/–^ mice were provided by T. W. Mak (University of Toronto, Toronto, Ontario, Canada), and *Nfil3*^–/–^ mice were bred in-house. C57BL/6 *Cd8*^–/–^ mice were provided by USTC. Six- to 12-week-old mice (male, if not otherwise specified) were used in this study. All mice were maintained in specific pathogen–free conditions according to the guidelines for experimental animals at the USTC.

### Tumor cell lines and challenge.

The MC38 cell line was a gift from Yangxin Fu (Southwestern Medical Center, University of Texas, Houston, Texas, USA). The MC38-Luc cell line was generated by infection with firefly luciferase–carrying lentivirus in our laboratory at USTC. The B16-F10 cell line was purchased from the cell bank of the Chinese Academy of Sciences (Shanghai, China). The Hepa1-6 cell line was purchased from Procell. All cell lines tested negative for mycoplasma contamination. The cells were cultured in DMEM (HyClone) supplemented with 10% FBS (Gibco) and 1× penicillin/streptomycin (Solarbio) at 37°C under 5% CO_2_ with normoxia. For experimental liver metastasis models, MC38 or MC38-Luc tumor cells (2 × 10^5^ cells/mouse) or B16-F10 tumor cells (1 × 10^5^ cells/mouse) were i.s. challenged, followed by splenectomy 3 minutes after injection. For the orthotopic liver cancer model, Hepa1-6 tumor cells (3 × 10^6^ cells/mouse) were challenged into the left lobe of the liver.

### In vivo antibody treatment.

The anti-FGL1 mAb (clone 177R4) was purchased from BioXCell, and the control rat IgG was purified in-house from rat serum. To explore the role of FGL1 under normal physiological conditions, WT mice were injected i.p. with anti-FGL1 mAb (200 μg) or control IgG (200 μg) on days 0 and 2, and then, on day 3, the livers and spleens were harvested for flow cytometry. For preventive treatment, mice were pretreated with anti-FGL1 mAb (200 μg) or control IgG (200 μg) 1 day before tumor cell challenge and then once every 4 days for a total of 4 times. For therapeutic treatment, MC38 tumor–bearing mice were treated with anti-FGL1 mAb (250 μg) or control IgG (250 μg) 4 days after tumor cell challenge, once every 4 days 4 times; and B16-F10 tumor–bearing mice were treated with anti-FGL1 mAb (200 μg) or control IgG (200 μg) 3 days after tumor cell challenge, once every 3 days, for a total of 5 times. To deplete NK cells, mice were injected i.p. with anti-ASGM1 (FUJIFILM Wako) (50 μL) once every 4 days 5 times at the beginning of tumor cell challenge. Mice were sacrificed at the indicated time points for study or investigated for survival.

### Bioluminescence imaging.

Bioluminescence imaging and data analysis for photon flux generated by MC38-Luc liver metastatic tumors were performed using the in vivo imaging system (IVIS) spectrum (PerkinElmer). Mice were injected (150 mg/kg body weight, i.p.) with d-Luciferin (Gold Biotechnology), fully anesthetized with isoflurane (RWD), and then placed into the IVIS imaging chamber. After 15 minutes of d-Luciferin injection, images were collected. Photons emitted from the tumor were quantified using Living Image Software (PerkinElmer).

### MNC isolation.

The liver samples were pressed through a 200-gauge mesh, and liver MNCs were collected after density gradient centrifugation in 40%–70% (*v/v*) Percoll solution (GE Healthcare, now Cytiva). The spleen samples were homogenized gently, and splenocytes were collected following the lysis of erythrocytes using red cell lysis buffer (Solarbio).

### Adoptive transfer of CD8^+^ T cells.

Hepatic MNCs from WT mice were isolated, and then CD8^+^ T cells were sorted by using mouse CD8α^+^ T isolation kit (Miltenyi Biotec). Then 2 × 10^5^ CD8^+^ T cells were i.v. transferred into *Cd8*^–/–^ mice 3 times (once a week), starting 1 day before tumor cell challenge.

### In vitro T and NK cell function assay.

For the T cell function assay, 2.5 μg/mL anti-CD3 mAb (BD Biosciences) and 1.25 μg/mL anti-CD28 (BD Biosciences) were precoated in 96-well flat plates, and hepatic MNCs from WT mice were isolated and added to 96-well flat plates (3 × 10^5^/well). For the NK cell function assay, hepatic NK cells from WT mice were sorted with a mouse NK cell isolation kit (Miltenyi Biotec), added to 96-well flat plates (5 × 10^4^/well), and then stimulated with 10 ng/mL IL-12 (Thermo Fisher Scientific), 50 ng/mL IL-15 (Thermo Fisher Scientific), and 10 ng/mL IL-18 (Absin). The cells were cultured at 37°C in the presence of 50 ng/mL recombinant mouse FGL1 protein (Sino Biological), 1 μg/mL anti–LAG-3 mAb (clone C9B7W) (BioXCell), or 1 μg/mL control IgG (purified in-house from rat serum) for 3 days. IFN-γ levels in the supernatant were detected by using a mouse IFN-γ–precoated ELISA kit (DAKEWE).

### Real-time PCR.

Total mRNA was extracted using TRIzol Reagent (Invitrogen). cDNAs were synthesized using an M-MLV Reverse Transcriptase kit (Invitrogen) according to the manufacturer’s protocol. Real-time PCR amplification was performed in 96-well optical reaction plates using SYBR Green Supermix (Takara) with the indicated primers. The primers used were as follows: *m*β*-actin*-F, GGCTGTATTCCCCTCCATCG; *m*β*-actin*-R, CCAGTTGGTAACAATGCCATGT; *mFgl1*-F, CATTGCTCTGATGATGGGAA; and *mFgl-1*-R, GCAAGAGCTGTGCAATCATG. Relative quantification of gene expression was calculated by using the comparative threshold cycle value method (2^-ΔΔCq^). *Fgl1* mRNA expression was normalized to β*-actin* expression.

### Flow cytometry analysis.

The antibodies used for flow cytometry analysis are detailed in Supplemental Table 1. The cell surface molecules were stained with fluorescence-labeled mAbs after the Fc receptors were blocked with rat serum. For intracellular staining of cytokines, cells were stimulated with 1 mg/mL ionomycin (MilliporeSigma) and 30 ng/mL phorbol myristate acetate (MilliporeSigma) for 4 hours, and 2 mg/mL monensin (MilliporeSigma) was added at the start of stimulation. After surface molecule staining, the cells were fixed and permeabilized by using a FoxP3/Transcription Factor Buffer (eBioscience) and then stained with mAbs against the intracellular molecules. Flow cytometry was performed on the LSR Fortessa (BD Biosciences), and the data were analyzed with FlowJo software (Tree Star).

### Statistics.

Data were presented as the mean ± SEM, and statistical analysis was performed using Prism software (version 8.0.1; GraphPad). Comparisons between 2 sets of data were performed using 2-tailed unpaired Student’s *t* test. For statistical analysis of more than 2 groups, 1-way ANOVA followed by Tukey’s multiple comparisons or 2-way ANOVA followed by Holm-Šídák multiple comparisons test was used. The log-rank (Mantel-Cox) test was used for survival analysis. *P* < 0.05 was considered to indicate statistical significance.

### Study approval.

All experimental procedures involving mice were approved by the Ethics Committee of the USTC.

### Data availability.

Values for all data points in graphs are reported in the Supporting Data Values file.

## Author contributions

YC, RS, and ZT initiated and designed the research. FX performed all the experiments and analyzed and interpreted the results. YC, RS, and ZT wrote the manuscript. FX, HS, HP, and HW contributed to the discussion of the results. ZL provided the *Cd8^–/–^* mice.

## Supplementary Material

Supplemental data

Unedited blot and gel images

Supporting data values

## Version 1 07/08/2024

Electronic publication
