# Supplementary material for: Hepatocyte-derived FGL1 accelerates liver metastasis and tumor growth by inhibiting CD8+ T and NK cells
Source: JCI Insight. 2024 May 23;9(13):e173215. doi: 10.1172/jci.insight.173215 (PMC11383586; doi:10.1172/jci.insight.173215)
Supplement: Supplemental data [file jciinsight-9-173215-s012.pdf]

Supplemental Materials and Methods

Generation and identification of *Fgl1*-deficient mice

Fgl1-deficient mice were generated by a CRISPR/Cas9-mediated genome engineering strategy. Two guide RNAs directed Cas9 to excise exons 2-3 for a total of 1125 bp of the region, including the 250 bp coding sequence of *Fgl1* (sg1: GTTATTTCTAAGCTTCGATGGGG; sg2: CAGAAAGACCGTTAAGTACC-AGG), resulting in a functional knockout of the *Fgl1* gene. To identify the genotypes of the mice, total DNA was extracted using tail lysis buffer (prepared by Tris-HCl, EDTA, SDS, and NaCl) and proteinase K (TransGen Biotech, Beijing, China). PCR amplification was performed with the following primers: *Fgl1*-F, CACACCAGCCTCCTCTTAACAA; *Fgl1*-R, GTGAGTCATGTTGCTAATCATT GGA; and *Fgl1*-R', CCAACCTTCCCTTCCCATCATC. The size of the gene amplification products in the WT mice was 490 bp, and that in the *Fgl1*-deficient mice was 788 bp.

Immunoblotting

Total protein was extracted using RIPA lysis buffer (Servicebio, Wuhan, China) supplemented with protease inhibitor (Beyotime, Shanghai, China). Equal amounts of protein from the lysate were subjected to precast 4–20% Bis-Tris protein gels (GenScript Biotech, Nanjing, China) and separated proteins were transferred onto polyvinylidene difluoride (PVDF) membranes (Millipore, Bedford, MA, USA). The membranes were blocked with 5% (*w/v*) skim milk for 1 h at room temperature. After that, the membranes were incubated with primary antibodies against FGL1 (Proteintech, Chicago, IL, USA) and β-tubulin (Cell Signaling Technology, Boston, MA, USA) in 5% (*w/v*) skim milk in Tris-buffered saline containing 0.1% Tween-20 at 4°C overnight, and then incubated with anti-rabbit IgG and HRP-linked Ab (Cell Signaling Technology, Boston, MA, USA) for 1 h at room temperature. Chemiluminescence signals were captured with an Amersham Imager 600 (GE Healthcare, Chicago, IL, USA).

Histology

Liver samples were harvested and fixed in 4% paraformaldehyde (Servicebio, Wuhan, China) for at least 24 hours, then processed and embedded in paraffin, after which sections were prepared. The sections (3 μm) were dewaxed and rehydrated, followed by hematoxylin and eosin (HE) staining or immunohistochemical (IHC) staining. Sections for HE were photographed using a digital slide scanner (Pannoramic MIDI; 3DHISTECH, Budapest, Hungary) with CaseViewer software. For IHC staining, the sections were processed by heat-induced epitope retrieval in citric acid buffer (pH 6.0). An anti-FGL1 polyclonal antibody (Proteintech, Chicago, IL, USA) was used for immunostaining. After incubation with the primary antibody, the sections were incubated with the secondary antibody (Zhong Shan-Golden Bridge Biological Technology, Beijing, China) subsequently incubated with a 1× DAB reaction mixture (Cell Signaling Technology, Boston, MA, USA), and finally stained with hematoxylin. Sections for IHC were photographed using an optical microscope (ZEISS Axio Imager.A2, Jena, Germany).

Multiplex cytokine analysis

The serum of *Fgl1*$^{+/+}$ mice and *Fgl1*$^{-/-}$ mice was harvested, and cytokines (IL-1α, IL-6, IFN-γ and TNF-α) in the serum were detected with a Luminex 200 system (Luminex) by Univ-bio Company (Univ-bio, Shanghai, China).

Hepatocyte and nonparenchymal cell isolation

Livers were perfused with 0.5 mM EGTA solution (pH 7.2) and digested using Dulbecco's modified Eagle's medium (DMEM, HyClone, South Logan, UT, USA) supplemented with 0.075% (*w/v*) type I collagenase (Sigma–Aldrich, St. Louis, MO, USA) at 37°C. Isolated cells were resuspended in serum-free DMEM (HyClone, South Logan, UT, USA), and hepatocytes were collected following 60% (*v/v*) Percoll solution (GE Healthcare, Chicago, IL, USA) density gradient centrifugation. To obtain hepatic nonparenchymal cells (NPCs), livers were perfused with 0.5 mM EGTA solution (pH 7.2) and DMEM supplemented with 0.04% (*w/v*) type IV collagenase (Sigma–Aldrich, St. Louis, MO, USA) and digested with DMEM supplemented with 0.08% (*w/v*) type IV collagenase and DNase I (Sigma–Aldrich, St. Louis, MO, USA) at

37°C. Isolated cells were centrifuged followed by 40% (*v/v*) Percoll gradient density centrifugation to collect NPCs. Subsequently, hepatocytes and NPCs were subjected to mRNA extraction and real-time PCR.

Measurement of the serum levels of ALT and AST

Serum levels of alanine aminotransferase (ALT) and aspartate aminotransferase (AST) were measured using a diagnostic kit (DIRUI, Changchun, China) according to the manufacturer's protocol with a biochemical analyzer (Rayto, Shenzhen, China).

Cell proliferation assay

MC38, B16-F10, and Hepa1-6 cells were seeded into 96-well plates at 5,000 cells/well, cultured for 24h, and then treated with the indicated concentrations of recombinant mouse FGL1 protein (CUSABIO, Wuhan, China). The proliferative capacity of the cells was tested after 24 h by adding 10 μL CCK8 chemicals (GLPBIO, Montclair, CA, USA), and incubating at 37°C for 1.5 h, after which the optical density (OD) at 450 nm was detected.

Wound healing assay

To investigate the effect of FGL1 on the migration of MC38, B16-F10 and Hepa1-6 cells, wound healing assays were performed. The cell suspensions were seeded into 24-well plates at 3×10^5 cells/well. After culturing for 24 h, wounds were made by using a 1000 μL micropipette tip, and then the cells were treated with the indicated concentrations of recombinant mouse FGL1 protein (CUSABIO, Wuhan, China). The distance of the wounds was photographed after 0 and 24 h scratching using an optical microscope (Mshot, Guangzhou, China). The relative migration distance of cells at 24 h was compared to the corresponding distance at 0 h using ImageJ software (National Institutes of Health, Bethesda, MD, USA).

Database source and gene expression

The mRNA expression level of *Fgl1* in human tissues was acquired from the Genotype-Tissue Expression (GTEx) database (https://www.gtexportal.org/), and that in mouse tissues was acquired from the BioGPS database (http://biogps.org/). Graphs were generated using Prism software (version 8.0.1; GraphPad, San Diego, CA, USA).

Supplemental Table 1. Antibodies used for flow cytometry in this study

Fluorescence	Antibody	Company	Catalog	Clone
FITC	Anti-mouse CD69	BD Biosciences	553236	H1.2F3
FITC	Anti-mouse CD226	Biolegend	128803	10E5
FITC	Anti-mouse PD-1	eBioscience	11-9985	J43
FITC	Anti-mouse CD107a	BD Biosciences	553793	1D4B
Percp-CY5.5	Anti-mouse CD11b	Biolegend	101228	M1/70
Percp-CY5.5	Anti-mouse CD44	BD Biosciences	560570	IM7
Percp-CY5.5	Anti-mouse-B220	Biolegend	103236	RA3-6B2
Percp-CY5.5	Anti-mouse IFN-γ	Biolegend	505822	XMG1.2
Percp-CY5.5	Anti-mouse Foxp3	BD Biosciences	563902	R16-715
PE	Anti-mouse CD27	Biolegend	124210	LG.3A10
PE	Anti-mouse CD69	BD Biosciences	553237	H1.2F3
PE	Anti-mouse NKG2A	eBioscience	12-5897	16a11
PE	Anti-mouse MHCII	eBioscience	12-5321	M5/114.15.2
PE	Anti-mouse/human Granzyme B	Biolegend	372208	QA16A02
PE-Cy7	Anti-mouse CD49b	Biolegend	108922	DX5
APC	Anti-mouse NKG2D	eBioscience	17-5882	CX5
APC	Anti-mouse Siglec-H	Biolegend	129612	551
APC	Anti-mouse Helios	Biolegend	137222	22F6
Alexa 660	Anti-mouse Ki67	eBioscience	50-5698-82	SolA15
BV421	Anti-mouse NKG2D	BD Biosciences	562800	CX5
BV421	Anti-mouse CD11c	Biolegend	117330	N418
BV421	Anti-mouse TNF-α	Biolegend	506328	MP6-XT22
BV510	Anti-mouse CD4	BD Biosciences	563106	RM4-5
BV510	Anti-mouse CD45	BD Biosciences	563891	30-F11
BV605	Anti-mouse NK1.1	Biolegend	108740	PK136
BV786	Anti-mouse CD49a	BD Biosciences	740919	Ha31/8
BUV395	Anti-mouse CD3e	BD Biosciences	563565	145-2C11

Continued

Fluorescence	Antibody	Company	Catalog	Clone
BUV395	Anti-mouse TCR-β	BD Biosciences	742485	H57-597
BUV563	Anti-mouse CD4	BD Biosciences	565709	GK1.5
BUV737	Anti-mouse CD8α	BD Biosciences	564297	53-6.7

Supplemental Figure 1

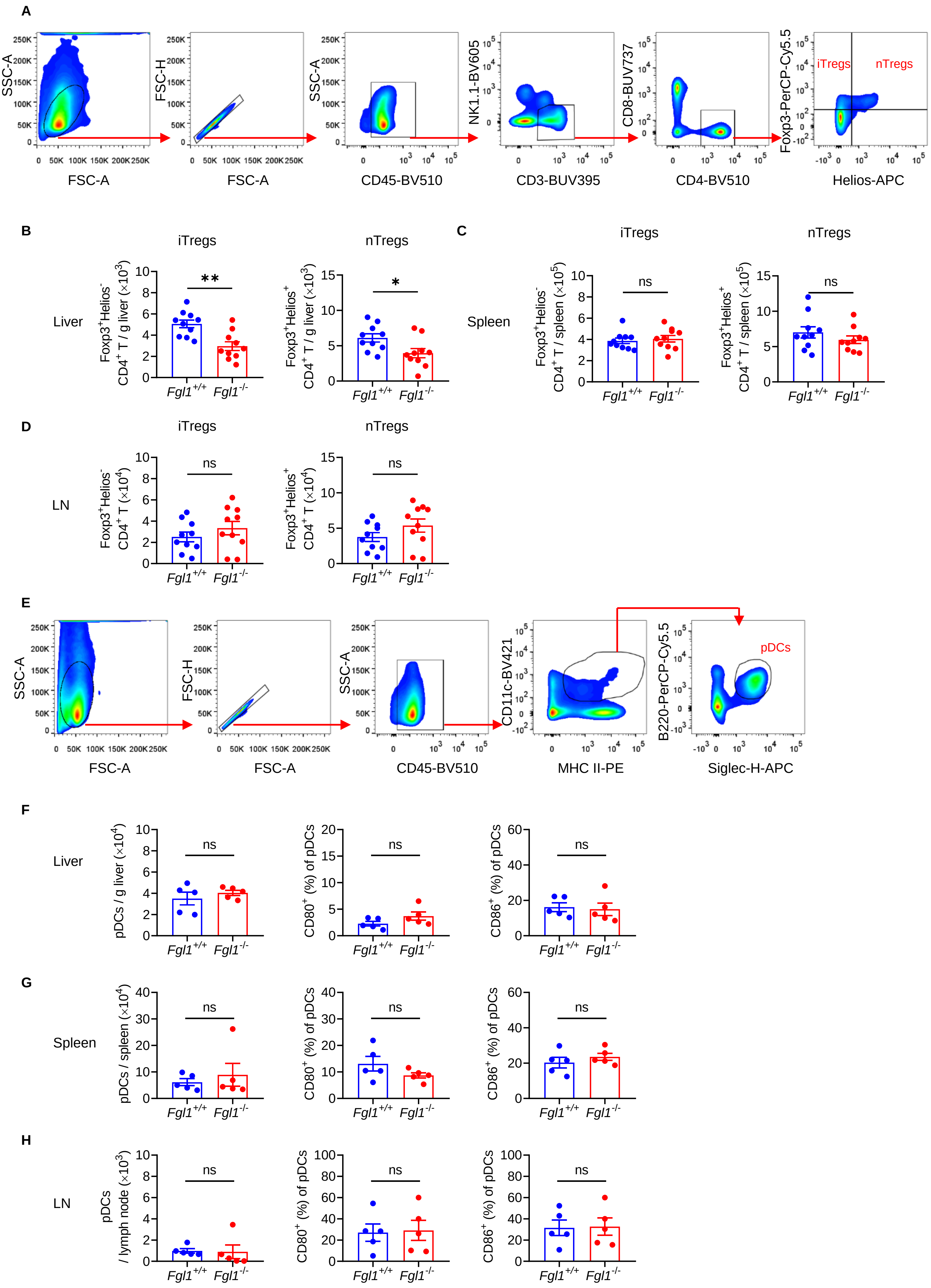

Supplemental Figure 1. Tregs and pDCs in the liver, spleen and lymph nodes of *Fgl1*-deficient mice compared with control mice. Livers, spleens and lymph nodes were harvested from *Fgl1*$^{+/+}$ and *Fgl1*$^{-/-}$ mice, and then MNCs were isolated and analyzed by flow cytometry. (**A**) Gating strategies for analysis of iTregs (Foxp3^{+}Helios^{-}CD4^{+} T cells) and nTregs (Foxp3^{+}Helios^{+}CD4^{+} T cells). Absolute numbers of iTregs and nTregs in the liver (**B**), spleen (**C**) and lymph node (**D**) of *Fgl1*$^{+/+}$ and *Fgl1*$^{-/-}$ mice (n = 10/group). Data are pooled from two independent experiments. (**E**) Gating strategies for analysis of plasmacytoid dendritic cells (pDCs, CD45^{+}CD11c^{+}MHCⅡ$^{+}$Siglec-H^{+}B220^{+} cells). Absolute numbers of pDCs (left) and frequencies of CD80 (middle) and CD86 (right) on pDCs in the liver (**F**), spleen (**G**) and lymph node (**H**) of *Fgl1*$^{+/+}$ and *Fgl1*$^{-/-}$ mice (n = 5/group). Representative of two independent experiments were shown. Comparisons were performed by using two-tailed unpaired Student's *t* test. Data are presented as the mean ± SEM (**B**-**D**, **F**-**H**). $^{**}P < 0.01$, $^{*}P < 0.05$. ns, not significant.

Supplemental Figure 2

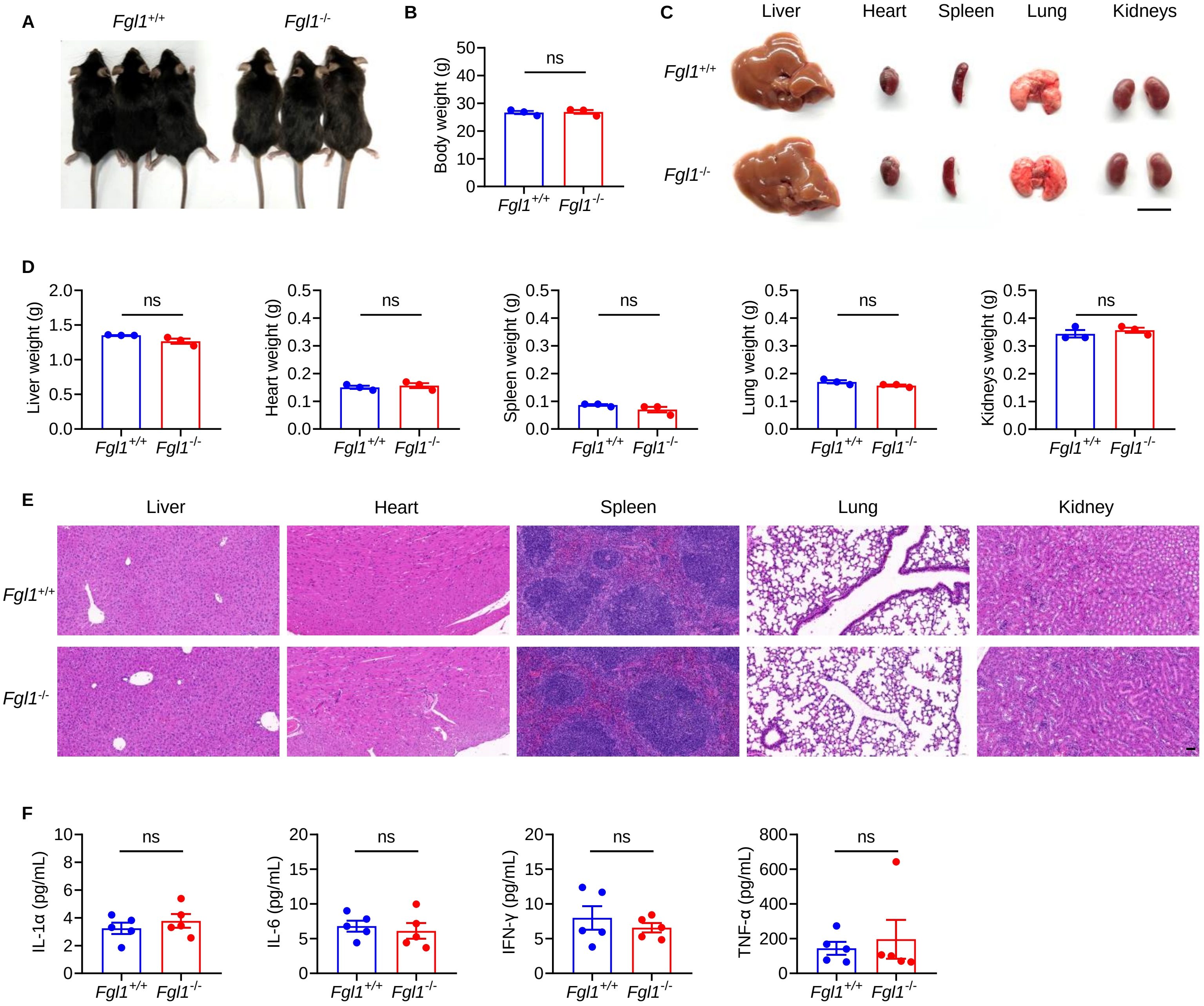

Supplemental Figure 2. No obvious changes are observed in adult *Fgl1*$^{-/-}$ mice compared with WT control mice. *Fgl1*$^{+/+}$ and *Fgl1*$^{-/-}$ mice were harvested, and the main organs were collected. (**A**) Representative photograph of *Fgl1*$^{+/+}$ and *Fgl1*$^{-/-}$ mice (n = 3/group). (**B**) Body weight of *Fgl1*$^{+/+}$ and *Fgl1*$^{-/-}$ mice (n = 3/group). (**C**) Representative photograph of liver, heart, spleen, lung and kidneys from *Fgl1*$^{+/+}$ and *Fgl1*$^{-/-}$ mice (Bar, 1 cm). (**D**) Weight of liver, heart, spleen, lung and kidneys from *Fgl1*$^{+/+}$ and *Fgl1*$^{-/-}$ mice (n = 3/group). (**E**) Representative histopathological images of liver, heart, spleen, lung and kidney from *Fgl1*$^{+/+}$ and *Fgl1*$^{-/-}$ mice (Bar, 50 μm). (**F**) The serum levels of IL-1α, IL-6, IFN-γ and TNF-α in *Fgl1*$^{+/+}$ and *Fgl1*$^{-/-}$ mice (n = 5/group). Comparisons were performed by using two-tailed unpaired Student's *t* test (**B**, **D**, **F**). Data are presented as the mean ± SEM (**B**, **D**, **F**). ns, not significant

Supplemental Figure 3

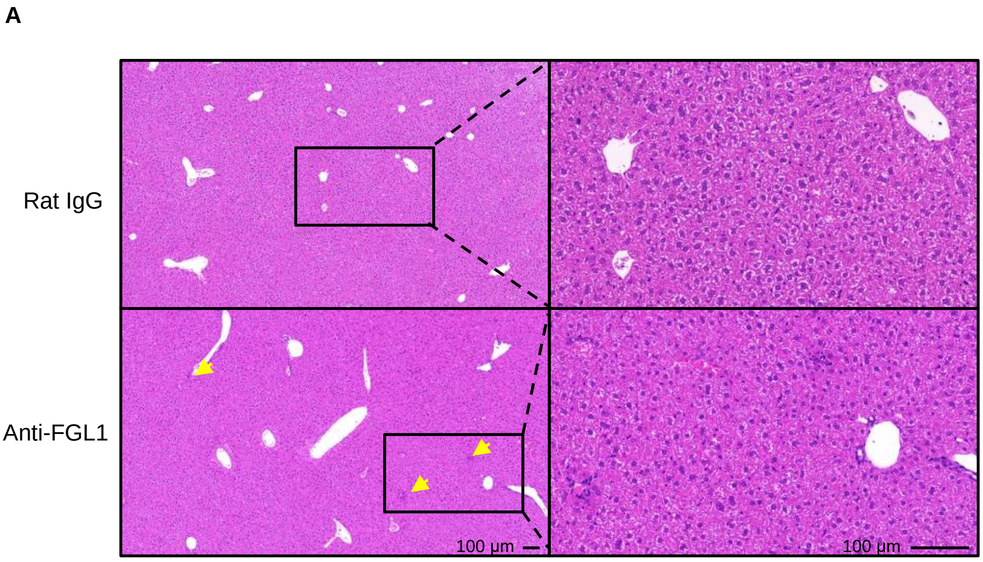

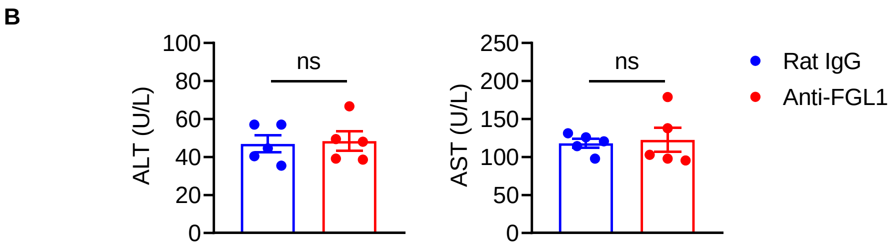

Supplemental Figure 3. Anti-FGL1 mAb treatment does not cause liver injury. WT mice were injected intraperitoneally (i.p.) with anti-FGL1 mAb (200 μg) or control rat IgG (200 μg) at day 0 and day 2, and then at day 3, serum and liver samples were harvested. (**A**) Representative histopathological images of livers. Bar, 100 μm. (**B**) The serum levels of ALT and AST (n = 5/group). Comparisons were performed by using two-tailed unpaired Student's *t* test (**B**). Data are presented as the mean ± SEM. ns, not significant.

Supplemental Figure 4

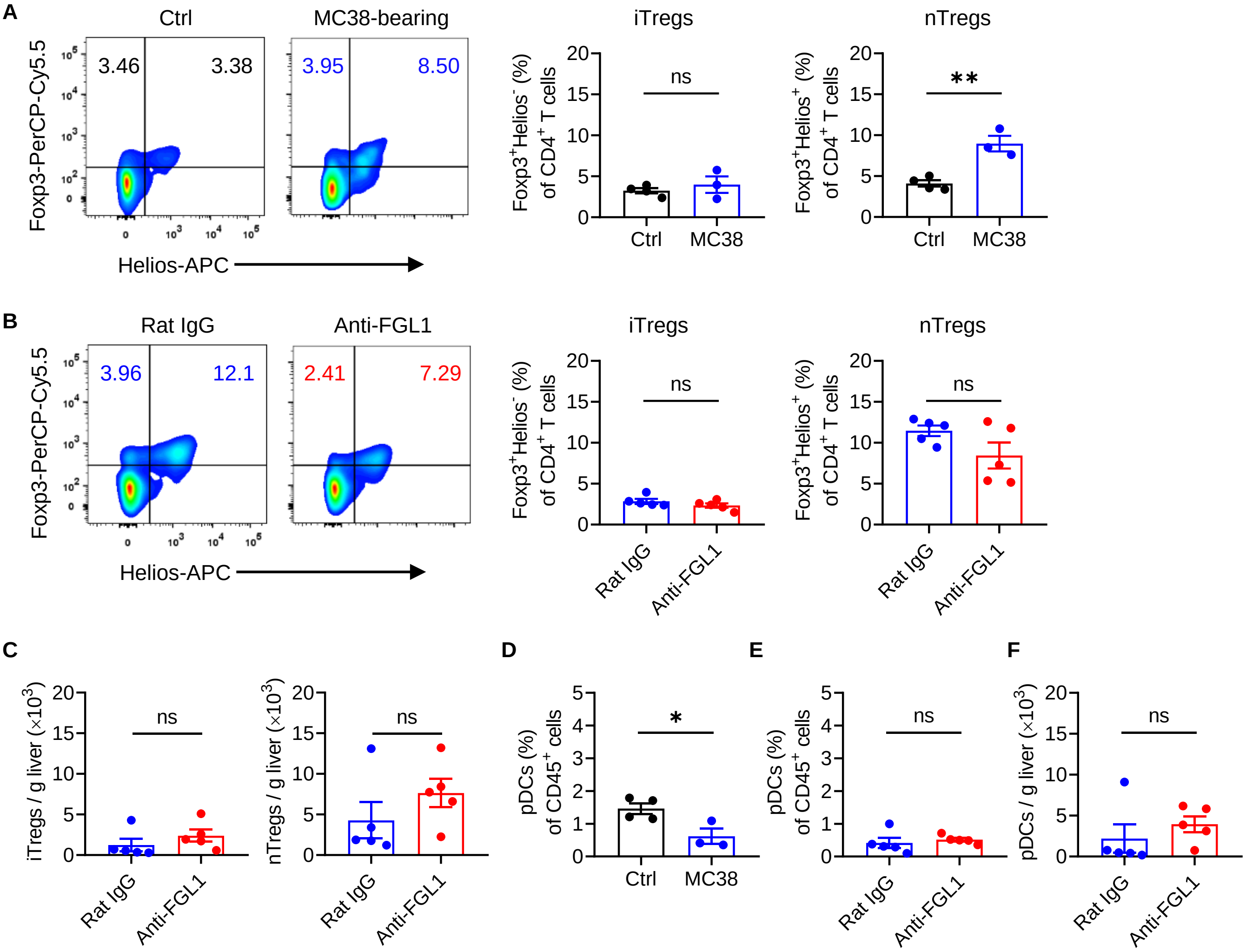

Supplemental Figure 4. Anti-FGL1 treatment affects Tregs and pDCs slightly. WT mice were intrasplenically (i.s.) challenged with 2 × 10^5 MC38 tumor cells. At 18 days after challenge, MNCs were isolated from livers and analyzed by flow cytometry analysis. (**A**) Representative plot (left) and frequency (right) of iTregs ($Foxp3^+Helios^-CD4^+$ T cells) and nTregs ($Foxp3^+Helios^+CD4^+$ T cells) in the liver of control mice and MC38-bearing mice (left). (n = 4 for the control group; n = 3 for the MC38-bearing group). MC38-challenged mice were treated intraperitoneally (i.p.) with 250 μg anti-FGL1 mAb or control rat IgG 4 days after tumor cell challenge 4 times (once every 4 days). (**B**) Representative plot (left) and frequency (right) and (**C**) absolute numbers of iTregs and nTregs in the liver on day 23 after MC38 challenge in anti-FGL1-treated mice and control mice (n = 5/group). (**D**) Frequency of plasmacytoid dendritic cells (pDCs, $CD45^+CD11c^+MHCII^+Siglec\text{-}H^+B220^+$ cells) in the liver of control mice (n = 4) and MC38-bearing mice (n = 3) on day 18 after challenge. (**E**) Frequency and (**F**) absolute numbers of pDCs in the liver on day 23 after MC38 challenge in anti-FGL1-treated mice and control mice (n = 5/group). Data are representative of two independent experiments. Comparisons were performed by using two-tailed unpaired Student's *t* test (**A**-**F**). Data are presented as the mean ± SEM. $^{**}P < 0.01$, $^{*}P < 0.05$. ns, not significant.

Supplemental Figure 5

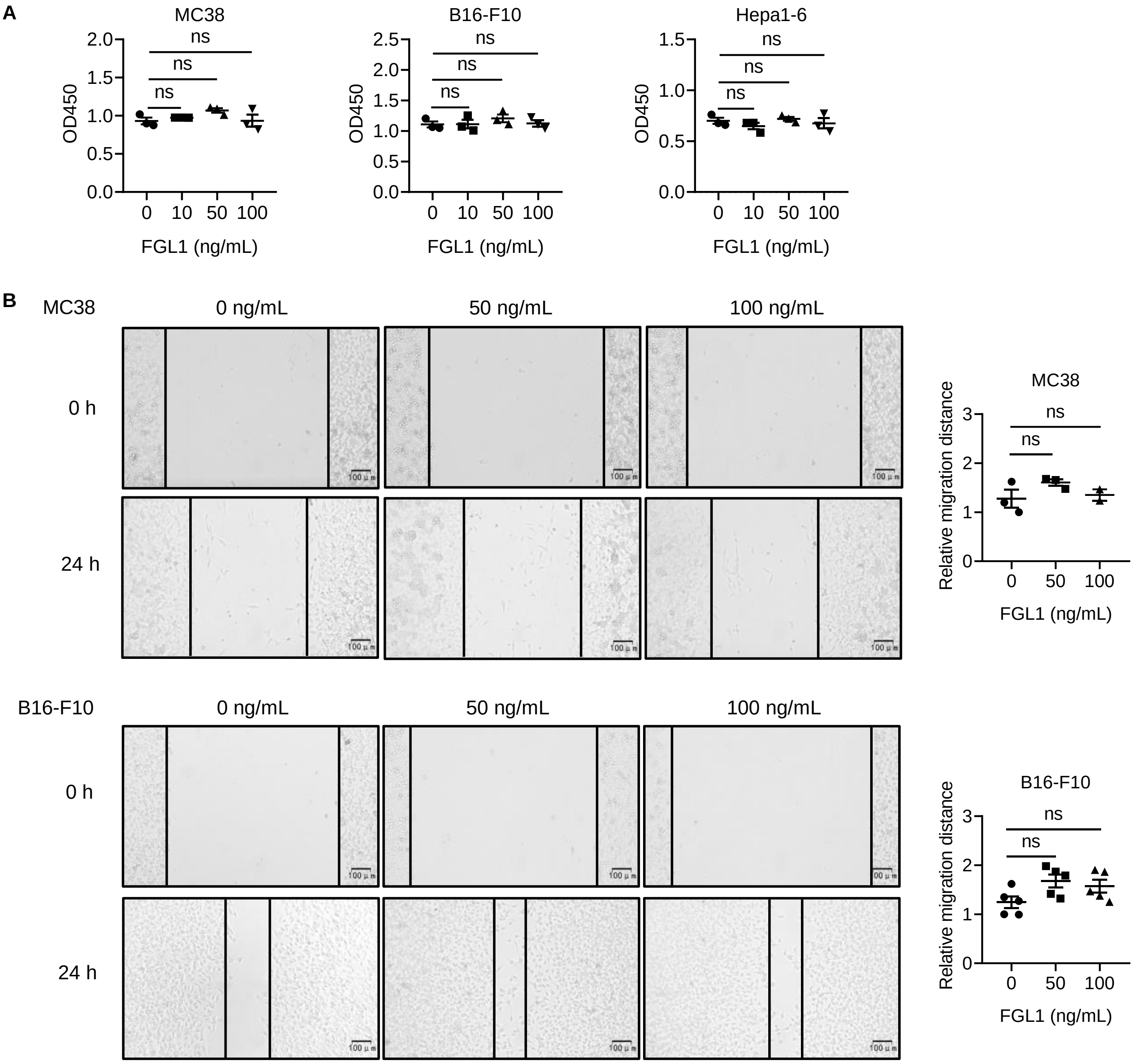

Supplemental Figure 5. Recombinant mouse FGL1 protein has no direct effects on the proliferation and migration of tumor cells. MC38, B16-F10 and Hepa1-6 tumor cells were treated with a series of concentrations of recombinant mouse FGL1 protein. (**A**) Proliferation of MC38, B16-F10 and Hepa1-6 tumor cells tested by Cell Counting Kit-8 assay. (**B**) Migration of MC38 and B16-F10 tumor cells assessed by wound healing assay. Representative photographs of tumor cells (left) and the relative migration distance of tumor cells (right) are shown. Comparisons were performed by using one-way ANOVA followed by Tukey's multiple comparisons test (**A**, **B**). Data are presented as the mean ± SEM. ns, not significant.
