# Supplementary material for: Hepatocyte-derived FGL1 accelerates liver metastasis and tumor growth by inhibiting CD8+ T and NK cells
Source: JCI Insight. 2024 May 23;9(13):e173215. doi: 10.1172/jci.insight.173215 (PMC11383586; doi:10.1172/jci.insight.173215)

Unedited blot for Figure 1C

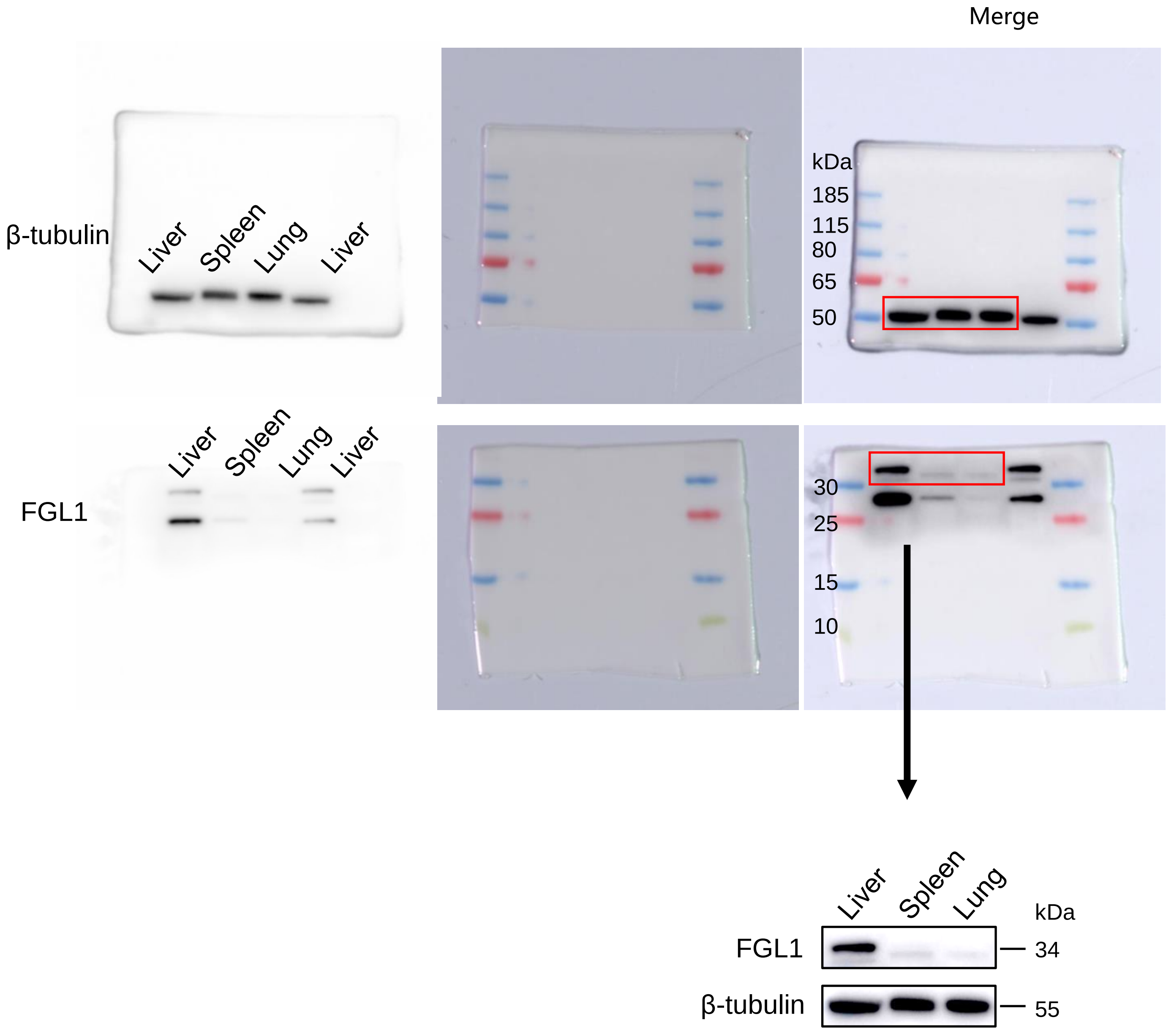

Full unedited gel for Figure 2B

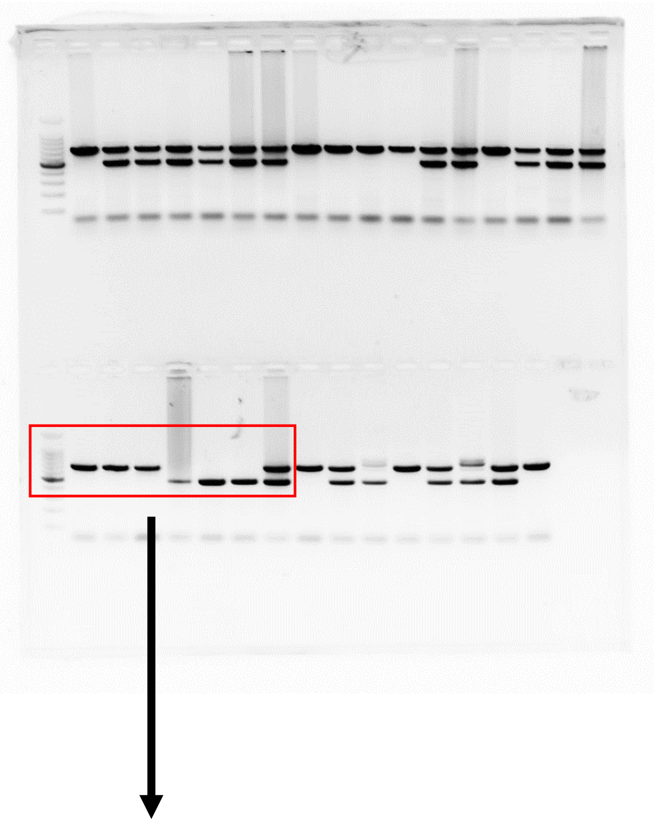

Mut 788bp
WT 490bp
Het 788bp & 490bp

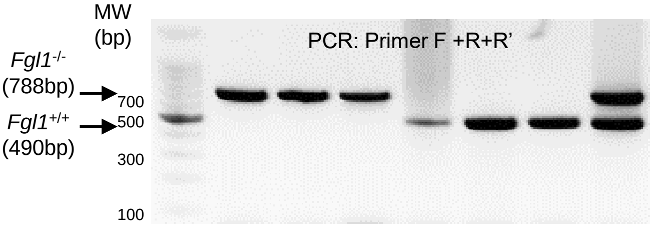

Unedited blot for Figure 2C

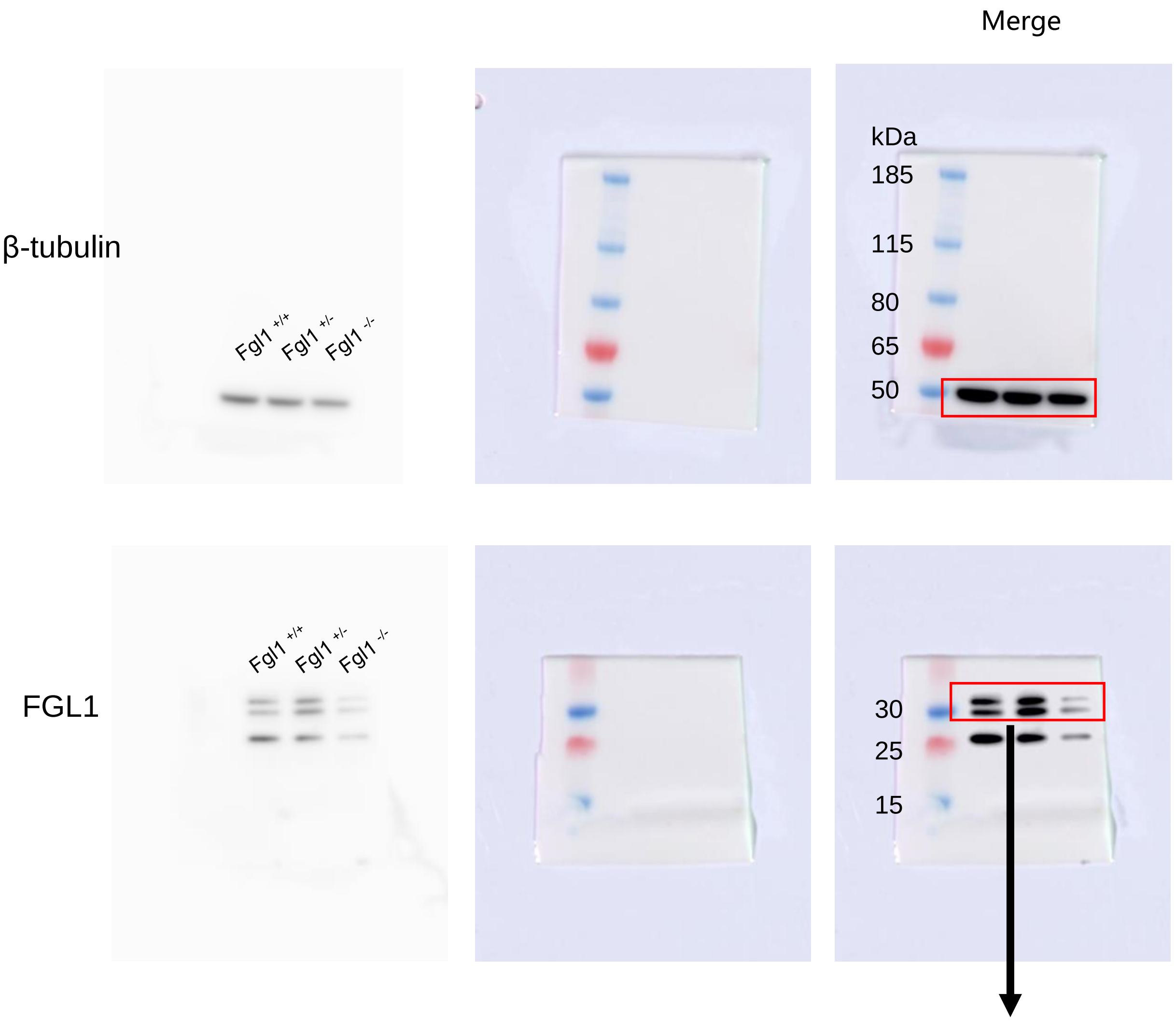

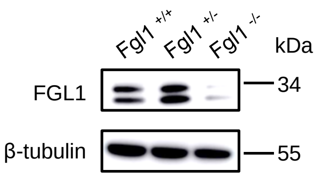

Full unedited gel for Figure 7B

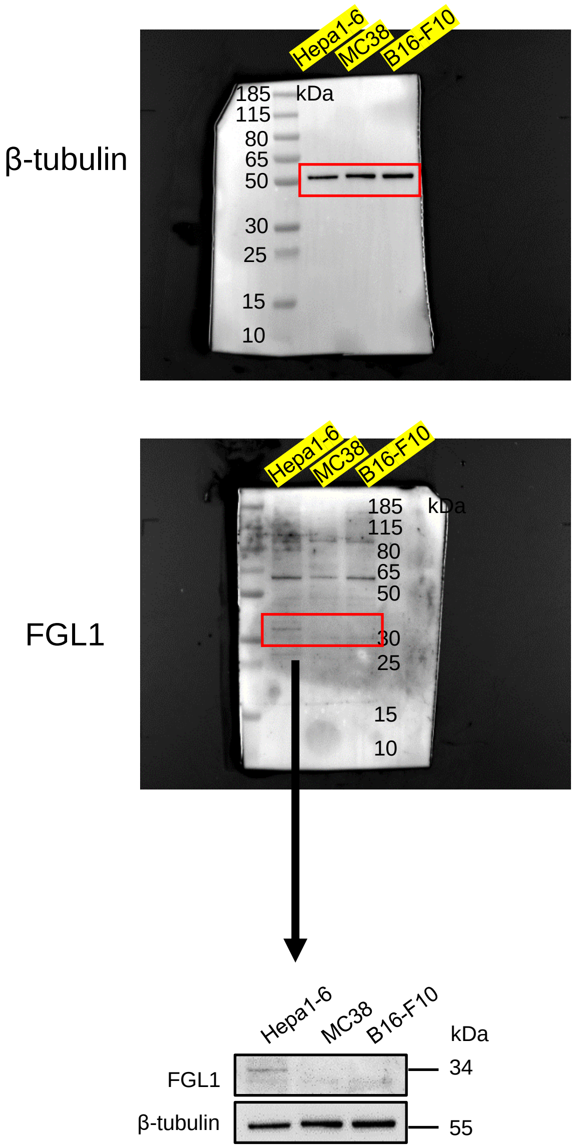

Supplement: Unedited blot and gel images [file jciinsight-9-173215-s013.pdf]
